# Identifying Patterns of Selection at Synonymous Sites in a Pseudomonas Phylogeny

**DOI:** 10.1093/gbe/evag241

**Published:** 2026-09-29

**Authors:** Luz Angela Alonso Morales, Susan F Bailey, Nicolas Rodrigue, Rees Kassen

**Affiliations:** Department of Biology, University of Ottawa, Ottawa, Ontario, Canada; Department of Biology, Clarkson University, Potsdam, NY, USA; Department of Biology, Institute of Biochemistry, and School of Mathematics and Statistics, Carleton University, Ottawa, Canada; Department of Biology, University of Ottawa, Ottawa, Ontario, Canada; Department of Biology, McGill University, Montreal, Quebec, Canada

**Keywords:** purifying selection, synonymous substitutions, substitution mappings, constrained evolution, substitution rate

## Abstract

A growing body of evidence suggests that synonymous substitutions, DNA sequence changes that do not alter the encoded amino acid and so should be selectively neutral, can be under strong selection. Yet our ability to identify putative non-neutral synonymous substitutions from comparative sequence data is limited. Here, we use Bayesian stochastic character mapping to contrast substitution rates and patterns for each position along two bacterial genes, followed by hierarchical clustering on principal components and consensus clustering to robustly identify synonymous sites showing signatures of selection. Specifically, we compare *gtsB* from *Pseudomonas fluorescens*, known from experimental studies to harbor synonymous mutations with strongly beneficial fitness effects, with a more conserved gene in the same operon, *gtsD*. We find that synonymous 4-fold degenerate sites can vary from highly constrained, characterized by low rates and constrained patterns of substitution comparable to neighboring nonsynonymous second codon positions, to weakly constrained, exhibiting high rates and diverse patterns of substitution indicative of weak purifying selection characteristic of nearly neutrally evolving sites. Highly constrained sites account for 8% and 7.5% of the 4-fold degenerate sites in *gtsB* and *gtsD*, respectively, and include some positions previously characterized through experiments as beneficial, suggesting they are sites that experience non-neutral selection. Patterns of codon usage, protein structure, or local sequence motifs do not explain the variation in substitution patterns between constrained and unconstrained sites. Combined, our site-specific approach suggests non-neutral synonymous mutations can occur at an appreciable frequency and may often contribute to adaptation.

Significance Synonymous substitutions, the replacement of one base without altering the encoded amino acid, are generally considered selectively neutral. However, experimental data suggest synonymous substitutions have a range of fitness effects and can play a significant role in evolution. Yet, it is still unknown whether specific synonymous positions in a gene are more likely to be non-neutral and, if that is the case, how often they contribute to adaptation. We analyzed patterns of substitution of third (synonymous) and second (nonsynonymous) positions of amino acids coded by four codons in Pseudomonas to identify signatures of selection at synonymous positions in two genes of an operon with evidence of adaptive synonymous mutations. Our results support the idea that synonymous substitutions are not always neutral. The strength of selection at synonymous sites varies across a gene sequence, with some sites likely experiencing strong purifying selection likely due to local structural, functional, or regulatory constraints.

## Introduction

Synonymous substitutions are thought to rarely, if ever, contribute to adaptive evolution because, despite a change to the DNA sequence, the resulting amino acid remains the same. Evidence supporting this assumption comes from the analysis of average substitution rates for nonsynonymous and synonymous sites within a codon. Changes to the second position and most changes to the first position of a codon are nonsynonymous and characterized by low substitution rates presumably because the altered amino acid is subject to purifying selection. Substitutions to the third position of the codon, by contrast, are very often synonymous, generating the same amino acid despite a change in underlying nucleotide, and typically exhibit high substitution rates (Bofkin and Goldman 2007; Arenas 2015). These substitution patterns are consistent with the idea that synonymous mutations are not “seen” by natural selection and so evolve more rapidly than the drastic molecular changes caused by nonsynonymous mutations (Kimura 1986). Further evidence supporting the claim that synonymous mutations are often neutral with respect to fitness comes from patterns of variation associated with codon redundancy: amino acids coded by six, four, or three codons typically have higher substitution rates than those coded by only two synonymous codons (Dufton 1983).

Nonetheless, the assumption that synonymous sites are neutral in the eyes of selection does not always hold. Multiple lines of evidence including codon usage bias (Grantham et al. 1980, 1981; Ikemura 1981; Bennetzen and Hall 1982; Ikemura 1985), the effects of synonymous mutations on transcription and translation (Sharp and Li 1986a, 1986b; Ermolaeva 2001; Rocha 2004; Chamary and Hurst 2005; Hershberg and Petrov 2008; Kudla et al. 2009; Saunders and Deane 2010; Sharp et al. 2010; Park et al. 2013; Presnyak et al. 2015; Hanson and Coller 2018; Zhang and Bebok 2022) and direct experimental estimates of fitness from both site-directed mutagenesis and laboratory evolution studies shows that synonymous sites can have both negative and positive fitness effects (Lind et al. 2010; Cuevas et al. 2012; Agashe et al. 2013, 2016; Bailey et al. 2014; Kershner et al. 2016; Kristofich et al. 2018; Lebeuf-Taylor et al. 2019). Taken together, these results suggest that synonymous sites can be under selection and even contribute to adaptation. What remains unclear is whether certain synonymous sites are more likely to be non-neutral and, if so, what mechanism(s) are responsible and how often they contribute to adaptation.

Efforts to address these issues with comparative DNA sequence data have been limited to date. The observation that synonymous sites are prone to higher rates of substitution compared with nonsynonymous sites, and so are likely to be neutral, led to the development of the widely used phylogenetic codon models to infer the mode and strength of selection in protein-coding sequences. These models analyze the ratios of nonsynonymous to synonymous substitution rates (*dN*/*dS*) under the assumption that synonymous sites are neutral and have a homogenous substitution rate (*dS*) across alignment sites (Goldman and Yang 1994; Muse and Gaut 1994; Murrell et al. 2015). Several models have been proposed to incorporate gene- or genome-wide patterns of selection on synonymous sites into phylogenetic analyses (Yang and Nielsen 2008; Zhou et al. 2010; Kubatko et al. 2016).

Recent phylogenetic approaches have demonstrated how *dN*/*dS* rate analyses underestimate the strength of purifying selection at synonymous sites and overestimate the frequency of positive diversifying selection acting on nonsynonymous positions in Bacteria (Wisotsky et al. 2020; Rahman et al. 2021), patterns that could be the result of varying rates of synonymous substitution across the genome. For example, Rahman et al. (2021) use a multiclass synonymous substitution (MSS) model applied to Enterobacterales genomes that allowed synonymous substitution rates to vary, classifying synonymous codons as either neutral or selected based on patterns of covariation in codon usage across genes. They found that, on average, sites classified in the “neutral” group had 25% higher synonymous substitution rates than those classified in the “selected” group, a result that shows how purifying selection can influence the substitution rate of synonymous codons across the genome. Furthermore, extensions of the MSS model have revealed substantial genome-wide heterogeneity in synonymous substitution rates across codon changes in genes from 12 *Drosophila* species, by inferring substitution rate classes directly from the data (Verdonk et al. 2025).

Substitution rates can vary substantially across sites within and between genes due to site-specific selective and biochemical constraints that generate unique patterns of accepted nucleotides or amino acids maintained by natural selection over long evolutionary timescales (Lartillot and Philippe 2004; Szánthó et al. 2023). Consequently, frameworks that account for variation in synonymous substitution rates using genome-wide patterns may not accurately capture the strength of selection acting on synonymous substitutions at specific positions in a gene or gene regions.

Here we take a distinct approach to inferring the identity and frequency of synonymous sites evolving non-neutrally by estimating the character history or the mapping of substitutions on a phylogeny, a powerful approach that allows the identification of specific features of the complete substitution process history over the phylogenetic tree (Laurin-Lemay and Rodrigue 2025). Bayesian stochastic character mapping (Nielsen 2002; Nielsen and Huelsenbeck 2002; Huelsenbeck et al. 2003) uses a substitution model to simulate, for each site, the detailed evolutionary history of substitutions, including the timing of each event along branches and their exact nature, conditional on the states in the alignment. In other words, the final states of the simulation reached along the terminal branches of the phylogeny match the observed states in the alignment. Obtaining a sample of such mappings is done by repeating this conditional simulation over a sample of parameter values from the posterior distribution. Because these mappings are conditional on the data and on posterior parameter values, they are often referred to as *posterior mappings*. In contrast, *posterior predictive mappings* are conditional on posterior parameter values but are not constrained to be compatible with the observed alignment; therefore, by contrasting both mappings, one can test specific evolutionary hypotheses and evaluate the absolute fit of a phylogenetic model to the data.

We examined patterns of substitutions at individual 4-fold degenerate sites (hereafter 4-fold synonymous sites) against second positions in the same codons (ie neighboring nonsynonymous changes), typically subject to strong purifying selection, in *gtsB* and *gtsD* across a phylogeny of 258 Pseudomonas strains. We have previously shown that individual synonymous changes can have strong beneficial fitness effects that can drive adaptive evolution in the same way as nonsynonymous substitutions in an evolution experiment with *Pseudomonas fluorescens*, independently of codon usage bias (Bailey et al. 2014, 2021; Lebeuf-Taylor et al. 2019). Bailey et al. (2014) identified two independently evolved beneficial 4-fold synonymous mutations in *gtsB*, a membrane-bound permease subunit of an ABC glucose transporter in Pseudomonas (*gtsABCD*, Fig. S1a). The two synonymous mutations improved fitness by increasing gene expression of *gtsB* and the other two downstream operon genes, *gtsC* and *gtsD*. Follow-up work in our lab showed that the distribution of fitness effects of beneficial synonymous and nonsynonymous mutations along *gtsB* are indistinguishable, and there was a positive correlation between the fitness advantage of synonymous mutations and their prevalence across a phylogeny of 77 related Pseudomonas species (Lebeuf-Taylor et al. 2019). The suggested mechanism responsible is the creation of “illicit” RNA polymerase binding sites that lead to increased expression of downstream genes, including *gtsD*, which is an ATP-binding subunit responsible for transporting glucose across the cell membrane and is considered a more conserved gene (Davidson et al. 2008).

Our Bayesian substitution mapping approach followed by hierarchical clustering on principal components (HCPC) and consensus clustering provides strong evidence that synonymous substitution rates vary across sites, indicating that synonymous changes are not always neutral. Our analysis revealed different classes of synonymous variants, ranging from “constrained” sites, characterized by low substitution rates and patterns (low diversity on acceptable base changes), to less constrained sites under “weak purifying selection,” which, in contrast, have higher substitution rates and more diverse patterns. Those synonymous positions identified as constrained represent eight and ten 4-fold synonymous sites in both genes and included two sites in *gtsB* where synonymous changes have previously been identified as beneficial experimentally (Bailey et al. 2014; Lebeuf-Taylor et al. 2019). We did not find evidence suggesting that codon usage bias, protein structure, or local sequence motifs explain these observations. Our findings highlight the relevance of considering site-specific selection constraints at synonymous positions and suggest that such constraints may contribute to adaptation in gene-specific contexts.

## Results

We characterized patterns of substitution (substitution rates, frequencies of all 12 possible base changes, transition rates, and transversion rates) at each nucleotide position of the genes *gtsB* and *gtsD* across a Pseudomonas species phylogeny (*N* = 258), focusing on identifying signatures of selection at synonymous sites. Our approach is based on Markov chain Monte Carlo simulations of distributions of posterior mappings (conditional on the observed data set) and posterior predictive mappings (expected/unconditioned on the observed data) of detailed substitution histories along the phylogeny. We contrast these distributions across site-degeneracy categories within genes as well as across genes. We then focus on the patterns of substitution of 4-fold synonymous sites and identified candidate 4-fold synonymous positions under strong purifying selection and tested for underlying mechanisms potentially driving differences in selection.

### High Substitution Rates at 4-Fold Synonymous Sites Drive Differences Between the *gtsB* and *gtsD* Genes of Pseudomonas

Each locus (nucleotide position) in the codons of the *gtsB* and *gtsD* genes of our collection of *Pseudomonas* species was classified into one of four degeneracy types: (i) 0-fold loci, is a codon position where any substitution would result in a change in amino acid, (ii) 2- and 3-fold loci, are codon positions where one or two out of three possible substitutions are synonymous, (iii) 4-fold loci, representing codons where all three changes at the third position are synonymous; and (iv) 6-fold loci, corresponding to the amino acids leucine, arginine, and serine that are encoded by six synonymous codons. Across degeneracy levels, we compared observed substitution rates at each nucleotide position against their posterior predictive distributions, calculating *Z*-scores, posterior predictive effect sizes (PPES) and posterior *P*-values (PPs) on the lower and upper tails of the distribution to evaluate the adequacy of the model on the data (Tables 1 and S1).

**Table 1:** Distribution of the mean number of observed and predicted substitutions across fold degeneracy levels at each site in *gtsB* and *gtsD*

Gene	Fold-degeneracy	Number of sites	Observed substitution rate^a^ (SD)	Predicted substitution rate^a^ (SD)	*Z*-score^b^ ± (SD)	PPES^c^ ± (SD)	Posterior *P*-value *lower* tail (PP)^d^ ± (SD)	Posterior *P*-value *upper* tail (PP)^d^ ± (SD)
*gtsB*	0	605	10.86 ± 24.19	11.43 ± 23.74	−0.17 ± 0.24	0.21 ± 0.16	0.67 ± 0.19	0.60 ± 0.13
	2	97	159.85 ± 127.13	160.57 ± 127.84	−0.01 ± 0.22	0.25 ± 0.18	0.52 ± 0.12	0.51 ± 0.12
	4	100	275.04 ± 166.38	273.48 ± 165.12	0.05 ± 0.29	0.26 ± 0.25	0.48 ± 0.14	0.54 ± 0.14
	6	107	131.07 ± 114.54	131.67 ± 114.5	−0.01 ± 0.3	0.28 ± 0.22	0.51 ± 0.15	0.52 ± 0.14
	Total	909	69.97 ± 122.3	70.32 ± 121.75	−0.11 ± 0.27	0.23 ± 0.18	0.61 ± 0.19	0.57 ± 0.14
*gtsD*	0	736	16.56 ± 33.96	17.07 ± 33.98	−0.14 ± 0.29	0.23 ± 0.23	0.66 ± 0.21	0.62 ± 0.15
	2	147	127.84 ± 84.1	128.3 ± 84.7	0 ± 0.27	0.25 ± 0.22	0.51 ± 0.13	0.52 ± 0.13
	4	132	174.73 ± 89.47	173.87 ± 87.21	0.03 ± 0.34	0.3 ± 0.24	0.49 ± 0.15	0.52 ± 0.15
	6	116	122.63 ± 94.71	123.28 ± 92.73	−0.05 ± 0.31	0.23 ± 0.22	0.52 ± 0.14	0.50 ± 0.14
	Total	1,131	60.36 ± 85.37	60.72 ± 84.75	−0.09 ± 0.31	0.24 ± 0.23	0.61 ± 0.2	0.58 ± 0.16

^a^The observed and predicted substitution rates corresponds to the average number ± SD of substitutions within each fold degeneracy, while the total mean corresponds to the weighted average across all fold degeneracy levels.

^b^ *Z*-scores represent the differences between observed and predicted substitution rates normalized by the standard deviation of the predictive distribution per position. Values shown are mean *Z*-scores ± SD within each fold degeneracy level. Individual nucleotide-level *Z*-scores are provided in Table S1.

^c^PPES (Doyle et al. 2015; Fabreti et al. 2024) represent the absolute differences between observed substitution rates and the median of the posterior predictive distribution of substitution rates per position, normalized by the standard deviation of the posterior predictive distribution. Values shown are mean PPES ± SD within each fold degeneracy level. Individual nucleotide-level PPES values are provided in Table S1.

^d^PPs represent the proportion of posterior predictive draws in which the predicted substitution rate either exceeded (upper tail) or fell below (lower tail) the observed substitution rate at each site (Bollback 2002). Values shown are means ± SD across nucleotide positions within each fold-degeneracy level. Individual nucleotide-level PP values are provided in Table S1.

Table 1 shows that in general, all degeneracy levels for both genes show no relevant differences in effect sizes between these two distributions (*Z*-scores min = −0.83, max = 1.55, and PPES min = 5.55 × 10^−4^, max = 1.66 in *gtsB*; and *Z*-scores min = −0.64, max = 2.28, and PPES min = 1.83 × 10^−4^, max = 2.47 in *gtsD*; see Table S1). Both lower and upper PPs are on average close to 0.5 and show effect sizes, *Z*-scores, and PPES < 2, consistent with the expected behavior of PPs under a well-fitting model (Fabreti et al. 2024). The only exception was a single nonsynonymous site showing modest evidence of underestimation in *gtsD* (PP upper tail = 0.03, *Z*-score = 2.28, and PPES = 2.47). Thus, our results show the model used here works well, capturing trends and patterns in the data.

Next, we compared the substitution rates between *gtsB* and *gtsD* across degeneracy levels (Fig. S1c) using a negative binomial regression to test whether the effect of gene on observed substitution patterns is the same across degeneracy level. We evaluated significance by comparing versions of the models with and without the interaction term using a likelihood-ratio test (LRT). Our results show that the interaction between gene and fold-degeneracy has a significant effect on the substitution rates (χ^2^ = 25.271, df = 3, *P* = 1.35 × 10^−5^). The significant differences are driven by the high number of substitutions at 4-fold synonymous sites in *gtsB* compared with all other categories (post hoc tests, *P* < 0.034, *P*-adjustment = FDR, Table S1), as well as the high number substitutions at 0-fold sites in *gtsD* compared with 0-fold sites in *gtsB* (post hoc test, *P* < 0.0001, *P*-adjustment = FDR, Table S2). Overall, these differences show that *gtsB* harbors more synonymous substitutions than *gtsD*, consistent with the idea that *gtsD* is under stronger purifying selection at synonymous sites than *gtsB*.

### Divergent Substitution Rates and Profiles Characterize the Evolution of Different Classes of 4-Fold Synonymous Sites in *gtsB* and *gtsD*

Given the rate of substitution on 4-fold synonymous sites differs for *gtsB* and *gtsD*, we next asked whether we could identify candidate synonymous sites under selection and any potential genomic features associated with this and perhaps driving variation in substitution rates. To do this, we performed HCPC on the 4-fold synonymous sites of *gtsB* and *gtsD* coupled with consensus clustering to assess clustering robustness separately (Fig. 1; Tables S3 and S4). We included seven active variables, the first three of which were calculated at each 4-fold site using the mappings sampled from the posterior distribution: substitution rate, transition rate, and transversion rate. The remaining four active variables represent the relative probability that the 4-fold synonymous site of a codon ends in A, T, C, or G. These values were derived from normalized codon probabilities calculated using position-specific substitution profiles within each codon. Including them as active variables allows the clustering to capture both substitution patterns and codon-ending composition at each site, enabling direct comparison with other biological variables included as supplementary variables.

**Fig. 1.**
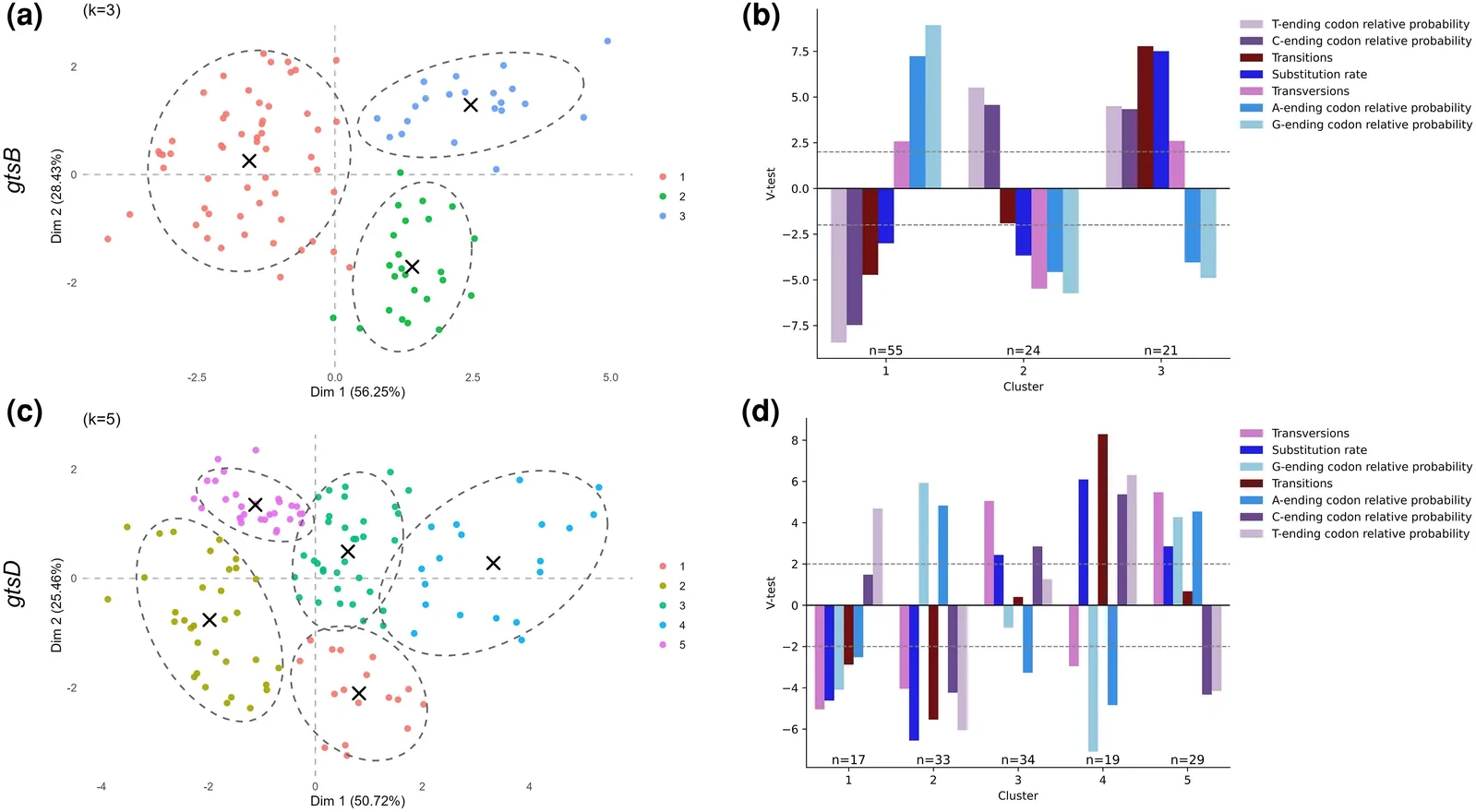
HCPC based on the Ward's criterion on 4-fold synonymous sites in *gtsB* (*n* = 100) and *gtsD* (*n* = 132). Clusters displayed were obtained using consensus clustering with a subsampling fraction of 0.8. Cluster assignments were highly consistent across the three subsampling fractions (Table S4; Figs. S3 and S5). The analysis involved seven quantitative active variables. a and c) Factor maps display each position within three (*gtsB*) and five (*gtsD*) clusters using the first two PCA dimensions which account for 84.68% and 76.18%, respectively, of the total variance. Dim 1 and Dim 2 correspond to PC1 and PC2, respectively. All variables are standardized. Ellipses indicate the visually identified clusters. b and d) Comparison of variables by cluster: The V-test scores represent a standardized deviation between the mean of each variable among positions in a cluster compared with the general average (Husson et al. 2017). Horizontal dotted lines indicate a cutline, where V-test scores equal to or >2 or equal or lesser than −2 are considered significant (*P* < 0.05).

The HCPC analysis, with cluster stability evaluated using consensus clustering (Fig. 1a and c), revealed that 4-fold synonymous sites in *gtsB* (*n* = 100) and *gtsD* (*n* = 132) formed three (*K* = 3) and five (*K* = 5) stable clusters, respectively, using the first two principal components (PCs). These first two PCs collectively explain 84.68% (*gtsB*) and 76.18% (*gtsD*) of the total variance. The number of PCs used for clustering was chosen via visual inspection of the proportion of variance plot and correspondent eigenvalues (Figs. S2 and S4) panels a and b. Figures S2c and S4c display the variable loadings for PC1 and PC2. We then chose the appropriate number of clusters for PCs 1 and 2 initially through visual inspection of the clustering dendrograms and Davies–Bouldin index plots (Fig. S2d and e for *gtsB* and Fig. S4d and e for *gtsD*). However, because the Davies–Bouldin index evaluates cluster separation but does not assess assignment stability, we evaluated clustering robustness in relation to variation in synonymous site composition within each gene using consensus clustering (Monti et al. 2003). For each gene, 4-fold synonymous sites were repeatedly subsampled (subsampling fractions of 0.7, 0.8, and 0.9 of sites per iteration), and clustering was evaluated for *K* ranging from 2 to 7. Cluster stability was evaluated using multiple complementary metrics, including the empirical cumulative distribution function (CDF) of consensus values, the change in CDF area across *K* (Δ*K*), the proportion of ambiguous clustering (PAC; Şenbabaoǧlu et al. 2014), mean within-cluster consensus, and site-level stability (for more details see Materials and Methods).

For *gtsB*, consensus clustering consistently supported *K* = 2 as the most stable partition; however, *K* = 3 retained high within-cluster consensus with only a modest reduction in stability, indicating additional consistent structure. Higher *K* values (*K* ≥ 4) showed reduced stability and signs of over-partitioning. We therefore retained *K* = 3 as the partition that best balances stability and resolution (Table S4; Fig. S3).

For *gtsD*, consensus clustering across subsampling fractions (0.7, 0.8, and 0.9) supported *K* = 5 as the most stable number of clusters. At this value, PAC was markedly reduced and overall stability was highest, with consistently high within-cluster consensus. Increasing *K* beyond five did not improve stability and led to the emergence of lower-consensus clusters. We therefore retained *K* = 5 as the most stable and reproducible partition (Table S4; Fig. S5).

Because cluster assignments in both genes were highly consistent across subsampling fractions, downstream analyses of variable contributions to each cluster were performed using the consensus results obtained with a subsampling fraction of 0.8. All seven variables contributed significantly to the analysis (*P* < 1 × 10^−4^), but the G- and T-ended codon frequency, transition and substitution rate are the variables better explained by the clustering (*η*^2^ ≥ 0.6) in both genes (Table S5).

Next, we described each of the clusters based on the contribution of the seven variables used in the analysis (Fig. 1b and d; Table S6). We employed a V-test (Husson et al. 2017) to find significance for each variable within a cluster. This test provides a standardized assessment of the deviation between the mean of each variable within a category (cluster) and the overall average, and whether that deviation is significant (V-test ≥ 2 or V-test ≤ −2, *P* < 0.05).

In *gtsB* (Fig. 1b), both clusters 1 (*n* = 55) and 2 (*n* = 24) are composed of sites with low substitution rates. However, sites in cluster 1 have high transversion rates, are enriched for G- and A-ending codons and depleted for T- and C-ending codons. In contrast, cluster 2 contains sites with the lowest substitution and transversion rates, enriched for T- and C-ending codons and depleted for G- and A-ending codons. Sites in cluster 3 (*n* = 21), despite showing similar codon-ending patterns to cluster 2, have the highest substitution rates and transition rates in the gene.

In *gtsD* (Fig. 1d), clusters 1 (*n* = 17) and 2 (*n* = 33) are composed of sites with low substitution and transition rates. Sites in cluster 1 have increased frequencies of T-ending codons and low frequencies for G- and A-ending codons, while cluster 2 comprises sites with the lowest substitution rates and is enriched for G- and A-ending codons and depleted for T- and C-ending codons. Sites in clusters 3 (*n* = 34), 4 (*n* = 19), and 5 (*n* = 29) have substitution rates that are higher than the overall mean. Clusters 3 and 5 are characterized by sites with high transversion rates but differ in their codon-ending relative probabilities: cluster 3 is mainly enriched for C-ending codons and depleted for A-ending codons, whereas cluster 5 shows the opposite pattern, with elevated G- and A-ending codon frequencies and reduced T- and C-ending codon frequencies. Sites in cluster 4 are the ones with the highest substitution and transition rates and are enriched for T- and C-ending codons and depleted for G- and A-ending codons.

Taken together, these findings suggest that 4-fold synonymous sites in both genes can be distinguished based on their substitution rates (ranging from low to high) and by the frequencies of synonymous codons (ending in T, G, C, or A). To better understand the nature of these clusters and evaluate if the frequencies of synonymous codons ending in T, G, C, or A and synonymous substitution rates are linked to patterns of codon usage, or to particular amino acids or gene regions, we also included as supplementary variables (variables that do not intervene in the construction of the HCPC) two quantitative metrics (Tables S3 and S5; Fig. S6a and b):

1. Relative synonymous codon usage “RSCU” of each analyzed gene, indicating observed codon frequency relative to equal usage among synonymous codons, and
2. The CAI adaptation weight (*w*), the genomic counterpart of RSCU, where *w* = 1 indicates the most frequently used codon within a synonymous group across the genome and values <1 indicate synonymous codons used less frequently relative to the most preferred one.

and three qualitative variables (Tables S3 and S7; Fig. S6c to f):

1. gene region where the site is located—we divided the genes into regions of 50 codons in length each to look general positional effects in the observed substitutional patterns,
2. protein topological organization—in *gtsB* this corresponds to inferred location of the encoded transmembrane helices (cytoplasm, membrane, and periplasm), while in *gtsD* this corresponds protein secondary structures (strand, helix, coil), and
3. amino acid.

Associations between cluster membership and qualitative supplementary variables were assessed using *χ*^2^ tests. In *gtsB*, amino acid (χ^2^ = 54.39, df = 8, *P* < 0.001) and gene region (χ^2^ = 24.45, df = 10, *P* = 0.006) were significantly associated with cluster membership. In *gtsD*, all three variables, amino acid (χ^2^ = 72.15, df = 16, *P* < 0.001), gene region (χ^2^ = 73.55, df = 28, *P* < 0.001), and protein topological organization (χ^2^ = 17.81, df = 8, *P* = 0.022), were significantly associated with cluster membership. Variable contributions and over- and underrepresented variables in each cluster were identified using the V-test (Husson et al. 2017), as described before.

Despite substantial heterogeneity in substitution rates across 4-fold synonymous sites in both genes, clusters with low and high substitution rates showed similar codon usage patterns as measured by RSCU and CAI weights (*w*) (Tables S3 and S6; Fig. S6a and b). For example, clusters 2 and 3 in *gtsB*, with a preference for T- and C-ending codons, and clusters 2 and 5 in *gtsD*, with a preference for G- and A-ending codons, showed contrasting substitution rates despite sharing similar codon usage profiles. This pattern suggests that variation in codon usage at 4-fold synonymous sites, regardless of whether due to translational selection or mutational bias, does not explain the observed differences in substitution rates among gene clusters.

Analysis of amino acid composition across clusters (Tables S3 and S7; Fig. S6c to f) indicates that biochemical differences in amino acid usage do not directly account for the observed substitution rate variation. For instance, glycine is abundant in clusters with both high and low substitution rates in both genes; it is overrepresented in the high substitution rate clusters (cluster 3 in *gtsB* and cluster 4 in *gtsD*) and also in the lowest substitution rate cluster in *gtsB* (cluster 2). In cluster 1 of *gtsD*, glycine occurs more frequently than the overall mean across clusters, although this difference is not significant (V-test < 2). Furthermore, glycine frequencies in *gtsD* are lowest in both cluster 2, which has the lowest substitution rates, and cluster 5, one of the fastest evolving clusters. These patterns suggest that amino acid composition alone does not determine substitution rate clusters.

Beyond amino acid composition, 4-fold synonymous sites from each substitution rate cluster were generally distributed throughout each gene, although certain regions showed significant (V-test ≥ 2) enrichment of particular rate classes. For instance, sites with the lowest substitution rates (cluster 2 in both genes) were predominantly found in the region comprising codons 101 to 150 in *gtsB*, while *gtsD* was enriched in regions 51 to 100 and 151 to 200 (Fig. S6c to f). The nonrandom spatial distribution of rate classes along each gene is consistent with structural or functional sequence constraints, potentially reflecting domains or sequence motifs under different selective constraints in both proteins.

Overall, we found a wide variety of active and supplementary variables with relevant contributions across different HCPC clusters, with no clear or consistent pattern to fully explain the differences in substitution rates across clusters. These findings suggest that various local and regional factors contribute to the observed variation in patterns of substitutions at 4-fold synonymous sites within these genes.

### Eight and Ten 4-Fold Synonymous Sites in *gtsB* and *gtsD* Display Signatures of Strong Purifying Selection

Is the variation in substitution rates among 4-fold synonymous sites driven by variation in selection, with some sites behaving neutrally and others experiencing strong purifying selection, or could it be the result of location-specific variation in mutation rates? (Chamary et al. 2006; Hershberg and Petrov 2010; Lee et al. 2012; Sung et al. 2015). Deciding between these alternatives is challenging but progress can be made by examining substitution patterns in the third position 4-fold synonymous sites with those at the neighboring second position (nonsynonymous) sites. We refer to these comparison sites as *neighboring nonsynonymous* sites. If local variation in mutation rates primarily drives substitution patterns (Sung et al. 2015), we would expect broadly similar substitution patterns between second- and third-position sites of the same codon. Marked differences between these positions are therefore more consistent with different selection constraints.

We first collected the following variables for both the neighboring nonsynonymous sites and the 4-fold synonymous sites: substitution rate, frequencies of all 12 possible base changes, transition rates and transversion rates (Table S8). This analysis directly compared the full substitution profiles at each position rather than codon-ending relative probabilities, since positions 2 and 3 within the same codon are not independent and a codon relative probability distribution cannot be generated. This approach captures position-specific substitution patterns and allows detection of constraint differences between these two codon positions.

We then applied HCPC analysis to the combined 4-fold synonymous and neighboring nonsynonymous sites data and performed hierarchical clustering coupled with consensus clustering, as before (Fig. 2; Tables S8 and S9). Since nonsynonymous sites tend to be subject to stronger purifying selection than synonymous sites, we assume that a 4-fold synonymous site that cluster with neighboring nonsynonymous sites is also likely subject to strong purifying selection. In contrast, we assume that 4-fold synonymous sites that group into clusters that are *not* dominated by neighboring nonsynonymous sites are likely subject to weak purifying selection.

**Fig. 2.**
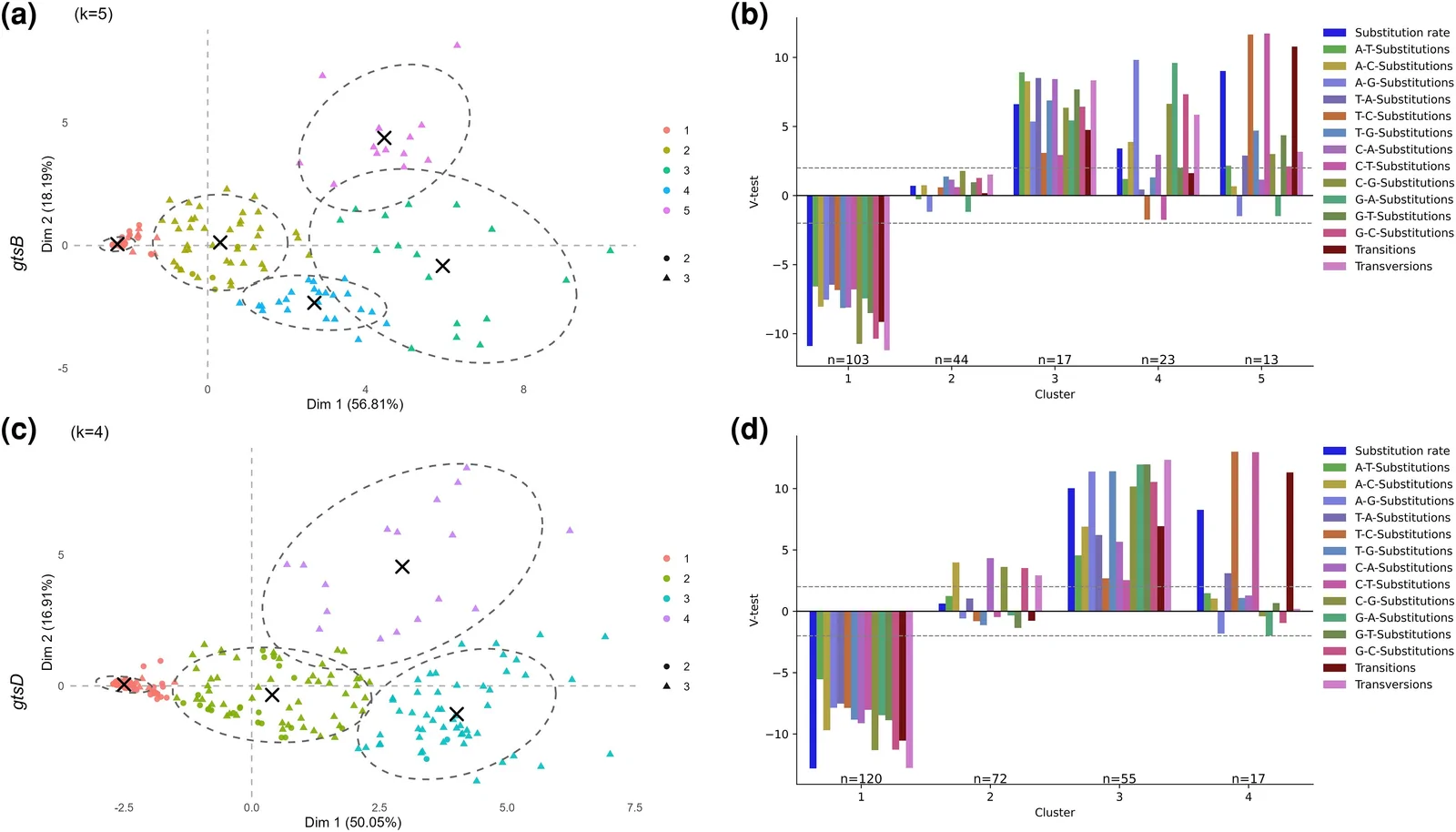
HCPC based on Ward's criterion on the neighboring nonsynonymous sites and 4-fold synonymous sites in *gtsB* (*n* = 200) and *gtsD* (*n* = 264). Clusters displayed were obtained using consensus clustering with a subsampling fraction of 0.8. Cluster assignments were highly consistent across the three subsampling fractions evaluated (see Table S9; Figs. S8 and S10). The analysis involved 15 quantitative active variables. a and c) Factor maps display each second and third position within five (*gtsB*) and four (*gtsD*) clusters using the first two PCA dimensions, which account for 75% and 67%, respectively, of the total variance. Dim 1 and Dim 2 correspond to PC1 and PC2, respectively. All variables are standardized. Colors represent different clusters. Triangles represent 4-fold synonymous sites, and circles represent neighboring nonsynonymous sites. Ellipses indicate the visually identified clusters. b to d) Comparison of variables by clusters (Table S11): The V-test represents a standardized deviation between the mean of each variable among positions in a cluster compared with the general average (Husson et al. 2017). Horizontal dotted lines indicate a cutline, where V-test scores equal to or >2 or equal or lesser than −2 are considered significant (*P* < 0.05).

Figure 2 displays the outcomes of the HCPC analysis of 4-fold synonymous sites and their neighboring nonsynonymous sites in the *gtsB* (panels a and b, *n* = 200) and *gtsD* (panels c and d, *n* = 264) genes. In Fig. 2a and c, five and four clusters in each gene are depicted with the first two PCs, which collectively explain 75% (*gtsB*) and 67% (*gtsD*) of the total variance. Detailed information on the corresponding variance plot, eigenvalues, and variable loadings can be found in Figs. S7 and S9, panels a, b, and c. As before, the number of clusters was initially evaluated using Ward's method, as illustrated by the dendrograms (Figs. S7d and S9d) and the corresponding Davies–Bouldin index plots (Figs. S6e and S7e). Because the Davies–Bouldin index assesses cluster separation but not assignment stability, we evaluated cluster robustness using consensus clustering, focusing on variation in nucleotide composition at 4-fold third positions and their associated second codon positions. For each site type, we repeatedly subsampled fractions of 0.7, 0.8, and 0.9 of sites per iteration and clustering was evaluated for *K* ranging from 2 to 7 (Table S9; Figs. S8 and S10).

For *gtsB*, consensus clustering indicated that *K* = 5 provided the most appropriate partition. Although *K* = 2 showed the highest overall stability, it produced a coarse division of the data. Additional structure became apparent at *K* = 4 (Δ*K* = 0.44), and assignment ambiguity was markedly reduced at *K* = 5 (PAC = 0.099). Increasing *K* beyond five yielded only marginal improvements in PAC and minimal gains in stability (Δ*K* = 0.015 at *K* = 6). Within-cluster consensus also remained consistently high at *K* = 5. We therefore retained this partition as the best balance between stability and structural resolution (Table S9; Fig. S8).

For *gtsD*, consensus clustering indicated that *K* = 4 provided the most appropriate partition. At *K* = 4, assignment ambiguity decreased substantially (PAC = 0.264), and overall stability reached its highest value (0.871), with consistently strong within-cluster consensus. Although PAC declined slightly at higher *K* values, the improvement was modest and coincided with the emergence of lower-consensus clusters, suggesting that increasingly smaller groups were being formed. Beyond *K* = 4, additional gains in cluster structure were minimal. We therefore retained *K* = 4 as the partition that best balances stability and structural resolution (Table S9; Fig. S10).

Because cluster assignments were stable across subsampling fractions in both genes, variable contribution analyses were based on the consensus partition obtained with a subsampling fraction of 0.8. All variables contributed significantly to the analysis (*P* < 1 × 10^−8^), but the substitution rate, as well as the transition and transversion rates, are the variables that are better explained by the clustering in both genes (*η*^2^ > 0.83, Table S10).

In both genes, we find the overwhelming majority of nonsynonymous neighboring sites group into a single cluster, cluster 1, that includes 95% of the nonsynonymous neighboring sites (*n* = 95; Fig. 2a) in *gtsB* and 83.3% of the nonsynonymous neighboring sites (*n* = 110; Fig. 2c) in *gtsD*. These two clusters likely capture characteristics of sites under strong purifying selection. In support of this inference, all variables in these two clusters have significantly lower values than average (Fig. 2b and d; V-test, *P* < 0.05, Table S11), a pattern consistent with sites undergoing minimal evolution. Within these “strong purifying selection” clusters, we also see eight 4-fold synonymous sites in *gtsB* (8%) and ten 4-fold synonymous sites in *gtsD* (7.57%) (Table S8) that remained stable in the constrained cluster across *K* = 2 to *K* = 7 and across all three resampling fractions, suggesting that these particular synonymous sites are evolutionarily constrained in similar ways to the nonsynonymous neighboring sites. In other words, these 4-fold synonymous sites are likely non-neutral and experiencing strong purifying selection.

Shannon entropy analysis (Shannon 1948) provided additional support for differences in patterns of substitution among site-type categories, including identified constrained 4-fold synonymous sites, weakly constrained 4-fold synonymous sites (sites not assigned to cluster 1), and neighboring nonsynonymous sites. The Shannon entropy analysis captures how substitutions at each site are distributed across the 12 possible nucleotide changes, rather than overall substitution rates. Consistently, contrasts among site-type categories support variation in selective constraints.

For *gtsB*, normalized substitution entropy differed among site types when evaluated using a linear model (with robust standard errors). Less constrained 4-fold synonymous sites exhibited higher entropy than constrained 4-fold synonymous sites (Δ = 0.134, 95% confidence interval, CI [0.041, 0.228], Holm-adjusted *P* = 0.03) and neighboring nonsynonymous positions (Δ = 0.209, 95% CI [0.157, 0.262], Holm-adjusted *P* < 0.001). Constrained 4-fold synonymous sites showed entropy comparable to neighboring nonsynonymous-positions (Δ = 0.074, 95% CI [−0.021, 0.171], Holm-adjusted *P* = 0.173). These results indicate reduced substitution diversity at constrained 4-fold synonymous sites in this gene.

In *gtsD*, less constrained 4-fold synonymous sites exhibited significantly higher entropy than neighboring nonsynonymous sites (Δ = 0.138, 95% CI [0.092, 0.184], Holm-adjusted *P* < 0.001). When compared with constrained 4-fold synonymous sites, however, they displayed only modest differences that did not reach statistical significance (Δ = 0.05, 95% CI [−0.026, 0.129], Holm-adjusted *P* = 0.30). Constrained 4-fold synonymous sites again showed entropy comparable to neighboring nonsynonymous sites (Δ = 0.086, 95% CI [−0.006, 0.168], Holm-adjusted *P* = 0.159). This weaker separation between constrained and less constrained 4-fold synonymous sites is consistent with the reduced substitution heterogeneity observed in this gene.

Interestingly, in a closer look at the group of the eight 4-fold synonymous sites in *gtsB* from the “strong purifying selection” cluster we recover synonymous sites of two codons previously identified in an evolution experiment as being under selection: codons 10 and 38. These two codons acquired beneficial mutations in independently evolving populations of *P. fluorescens*, as reported by Bailey et al. (2014). In one population, the synonymous mutation G38G (GGC→GGT) represented the most advantageous synonymous mutation, whereas in another population, the nonsynonymous mutation A10T (GCC→ACC) significantly improved fitness. Follow up work by Lebeuf-Taylor et al. (2019) demonstrated via site-directed mutagenesis that a synonymous mutation at codon 10 (A10A, GCC → GCT) also led to a significant fitness improvement under the same experimental conditions, supporting that these two synonymous sites are under positive selection under the tested conditions.

Our results provide strong support for the idea that synonymous sites are not always universally neutral. Rather, 4-fold synonymous sites can harbor distinct “classes” of synonymous substitutions based on their patterns of substitution. Many of these 4-fold synonymous sites have evolutionary characteristics indistinguishable from second-position nonsynonymous sites, with the potential for mutations at these sites to be beneficial, deleterious, or neutral depending on the environmental context.

### Posterior Predictive Permutations Support that the Identified 4-Fold Synonymous Sites are Under Strong Purifying Selection

To determine whether individual 4-fold synonymous sites within each gene had unusually low substitution rates and profiles relative to null expectations, we compared the observed number of constrained 4-fold synonymous sites to an empirical null distribution generated by randomly permuting neighboring nonsynonymous and 4-fold codon position labels within each posterior predictive mapping, with the assumption that these positions are interchangeable. In *gtsB*, only 8 third 4-fold synonymous sites were assigned to the constrained cluster, compared with a null mean of 54.1 ± 7.2 (SD) (min = 38, max = 75) across 1,100 permuted mappings (lower-tail empirical *P* < 0.001, *Z* = −6.4). Similarly, in *gtsD*, only 11 4-fold synonymous sites were identified as constrained compared with a null mean of 67.2 ± 10.4 (SD) (min = 43, max = 95) across 976 permuted mappings (lower-tail empirical *P* < 0.001, *Z* = −5.4). In both genes the observed number of constrained sites fell well below the minimum value observed across all permuted mappings, indicating that the identified synonymous sites were constrained well beyond chance expectations (Figs. S11 and S12). Site-level empirical *P*-values confirmed that no permuted mapping assigned any candidate site to the constrained cluster, with all 8 sites in *gtsB* and all 11 sites in *gtsD* showing site-level *P* < 0.001. For *gtsD*, consensus clustering revealed that one site had unstable cluster assignment across resampling iterations and was excluded, yielding final sets of 8 and 10 well-supported candidate synonymous sites in *gtsB* and *gtsD*, respectively (Table S12).

### Observed Proportion of 4-Fold Synonymous Sites Under Purifying Selection Exceed Those Expected Under a Null Model

To further evaluate whether the observed proportion of 4-fold synonymous sites clustering with neighboring nonsynonymous sites deviates from what would be expected under neutral expectations, we simulated random codon alignments using a codon-based model with the same tree topology and gene length as *gtsB*, under uniform strong purifying selection (*ω* = 0.02) on nonsynonymous sites. Simulated alignments were analyzed using the same approach as the observed data. As before, a 4-fold synonymous site was considered under purifying selection if it remained stable in the constrained lowest substitution-rate/profile cluster across *K* = 2 to *K* = 7 and all three resampling fractions.

The observed proportion of 4-fold synonymous sites meeting these 2 conditions in *gtsB* (8%) exceeds the maximum proportion seen across all 39 null replicates, none of which contained a single 4-fold candidate site (Fig. S13, empirical *P* = 0.025). Furthermore, because synonymous sites evolve neutrally under the null while nonsynonymous sites are subjected to strong, uniform purifying selection, all neighboring nonsynonymous sites were consistently clustered together in the constrained cluster across replicates and fractions (Table S13), and *K* = 2 clusters were generally best supported by all five consensus clustering metrics (Table S14). This consistent clustering pattern indicates that our approach does not randomly group 4-fold synonymous sites with the strongly constrained nonsynonymous sites.

The cross-K-stability and multi-fraction filters helped control false positives. Figure S14 shows that at fraction 0.7, one replicate had a 4-fold synonymous site that stayed in the constrained cluster from *K* = 2 to *K* = 7, but this same site was assigned to other clusters at the other two resampling fractions, demonstrating the relevance of applying the fraction filter. Similarly, at fraction 0.9, another replicate had a 4-fold synonymous site group with all neighboring nonsynonymous sites, but only at *K* = 4 (Table S13); this pattern disappeared at other *K* values, highlighting the value of the *K*-stability filter. Together, these two examples demonstrate why stability over both *K* and resampling fractions is necessary for generating a potential list of 4-fold synonymous sites under purifying selection.

### Internal Promoter-like Motifs and Secondary mRNA Structure do not Fully Explain Constrained Evolution of Synonymous Variants in *gtsB* and *gtsD*

There are now a number of compelling experimental studies demonstrating how synonymous mutations change local sequence motifs to create novel binding sites for transcription or translation factors that modulate downstream gene expression (Gu et al. 2012; Agashe et al. 2016; Bajaj et al. 2023). The *gtsBCD* portion of the operon is one of these examples: synonymous mutations in *gtsB* create illicit RNA polymerase binding sites that lead to increased transcription of the downstream *gtsCD* genes (Bailey et al. 2014; Lebeuf-Taylor et al. 2019). These novel binding sites can have positive or negative effects on fitness and so, over evolutionary time, may drive the preferential use of certain codons to either avoid or enhance the presence of these motifs (Callens et al. 2021). Alternatively, these novel transcription or translation initiation sites may arise as a fortuitous consequence of mutation to local sequence motifs that have evolved for other reasons. Either way, it is conceivable that the constrained synonymous sites we observe in our analysis are part of sequence regulatory regions that have the potential to modulate the transcription of the *gtsBCD* part of the operon (Bailey et al. 2014; Lebeuf-Taylor et al. 2019).

To evaluate this idea, we first looked for internal promoter-like motifs using a *Pseudomonas aeruginosa*-specific σ70 promoter predictor that predicts transcription start sites (TSS) (Coppens et al. 2022). To date, all experimental evidence on the fitness effects of synonymous mutations in *gtsB* comes from *P. fluorescens* SBW25, so we used this strain as a reference and then looked for the frequency of internal promoter-like motifs in *gtsB* and *gtsD* across each strain in the phylogeny. In *gtsB*, we recovered significant predictions (*P* < 0.005) for 22 promoter-like motifs on the leading strand and 7 on the reverse strand of *P. fluorescens* SBW25 (Table S15). In *gtsD*, 12 promoter-like motifs are predicted on the leading and 14 on the reverse strands of *P. fluorescens* SBW25 (Table S15).

Across the phylogeny of *gtsB*, we found positions 79 to 128 (codons 25 to 43) and positions 355 to 508 (codons 118 to 169) contain the highest frequency of predicted TSS. While the majority of these motifs were found on the leading strand (Fig. S15a), we also found these motifs in the same region on the reverse strand (Fig. S15c). Predictions on *gtsD* are only found in high frequency in position 317 (codon 106) on the leading strand (Fig. S15b) and on the reverse strand at regions between positions 234 to 354 (codons 78 to 118) and 558 to 630 (codons 186 to 210) (Fig. S15d). These highly repeatable promoter-like motif regions overlap with 37.5% (*n* = 3) and 60% (*n* = 6) of the candidate synonymous sites under strong selection in *gtsB* and *gtsD*, respectively, but do not fully explain the presence of other constrained synonymous sites at other sequence regions. Thus, while some constrained synonymous sites may be the result of long-term selection for specific sequence motifs impacting transcription and translation initiation, it is more likely that internal regulatory binding regions arise as the fortuitous consequence of mutations to sequence motifs that have evolved for other reasons.

Translation efficiency and transcript stability can also be affected by mRNA stability, with changes in fitness being associated with both increases and decreases to mRNA folding energy (Kudla et al. 2009; Del Campo et al. 2015; Agashe et al. 2016). To evaluate the contribution of mRNA stability to substitution rates, we examined the minimum folding energy of each 4-fold synonymous site grouped in the “strong purifying selection” cluster for each possible synonymous alternative allele with respect to the reference sequence of *P. fluorescens* SBW25 for *gtsB* and *gtsD* (Fig. S16). We identified synonymous alleles in both genes that generate more stable structures, but also others that lead to less stable transcripts, and ones that were not significantly different from the reference. Therefore, as before, we are unable to draw any general conclusions regarding the contribution of mRNA stability to the variation in selection on synonymous sites in our collection.

## Discussion

Motivated by the growing body of experimental research showing that synonymous mutations can have non-neutral fitness effects, we developed and applied a novel computational approach to characterize substitution patterns across synonymous and nonsynonymous sites in a collection of gene sequences from the Pseudomonas group. The main difficulty in using comparative approaches to identify synonymous sites under selection is that the rate of substitution at a specific site, gene, or genome region is typically compared with a so-called “neutral reference” site or region that are often themselves synonymous sites. In other words, we tend to look for deviations from neutrality in substitution patterns against data that are assumed, a priori, to be neutral. The approach is therefore tautological and our inferences depend intimately on the *assumption*, rather than the demonstration, that a site evolves neutrally.

Nonetheless, in some instances this assumption can be justified. In eukaryotes that possess extensive unselected or weakly selected regions such as pseudogenes this approach has been used to provide evidence for strong purifying selection action on 4-fold synonymous sites. Approximately 22% of synonymous sites in *Drosophila* (Lawrie et al. 2013) and from 11% to ∼15% of synonymous sites in humans (Keightley and Halligan 2011; Savisaar and Hurst 2018) show evidence of being under strong purifying selection using this approach. This method is challenging to employ in bacterial genomes because they typically harbor a much lower proportion of noncoding DNA (McCutcheon and Moran 2011), many of which are small intergenic regions densely packed with regulatory sites (Mira et al. 2001) and so are likely to be under strong selection, making it difficult to identify with confidence genomic sites or regions that evolve neutrally.

To overcome this challenge, we used Bayesian stochastic character mapping followed by HCPC and consensus clustering to test if signatures of constrained selection were present at 4-fold synonymous sites when compared against its nonsynonymous neighbor site (the second position in the same codon) that is, presumably, experiencing strong purifying selection. We focused on sites in *gtsB*, a gene where previous experimental work showed some synonymous sites in *P. fluorescens* were strongly beneficial and contrasted patterns of substitution with *gtsD*, a more conserved gene in the same operon, from a diverse phylogeny of closely related Pseudomonas species. We identified eight and ten (8% and 7.57%) 4-fold positions in *gtsB* and *gtsD* displaying evolutionary patterns similar to the biased/restricted substitution patterns of their nonsynonymous neighboring sites expected to be under strong purifying selection. Evidently, non-neutral synonymous sites are present in these genes and, while they may not be common, are not so rare they could not occasionally contribute to adaptation.

An important assumption here is that mutation processes do not systematically differ between neighboring nonsynonymous and 4-fold synonymous sites within the same codon and, therefore, that these positions experience similar local mutational and selective pressures. Because mutation rates depend on local dinucleotide and trinucleotide context (Jones et al. 1987; Lee et al. 2012; Sung et al. 2015), it is possible these positions may still experience different underlying mutation rates even in the absence of selection. Thus, although the observed differences in substitution rates and patterns between neighboring nonsynonymous and 4-fold synonymous sites are consistent with there being distinct selective constraints on some 4-fold synonymous sites, contributions from context-dependent mutation cannot be completely ruled out.

This caveat notwithstanding, additional evidence points to selection, rather than mutation bias, as the best explanation for our results. More than 80% of neighboring sites clustered together in both genes, and their entropy, reflecting the distribution of substitution types, was lower and significantly different from that of less constrained 4-fold synonymous sites. These findings suggest that neighboring sites’ substitution patterns are relatively uniform and unlikely to account for the observed differences through mutation bias alone. To further test this, we compared the observed candidate sites to each gene's own posterior predictive null, which is conservative by construction, since it reflects the same model complexity as the real data. Using this null, we found that the observed proportion of constrained 4-fold synonymous sites in each gene was rare compared with chance expectations, with null means of 54.1 (*gtsB*) and 67.2 (*gtsD*). At the level of individual sites, empirical *P*-values further confirmed that no permuted mapping ever assigned any candidate site to the constrained cluster.

Consistent with these findings, our second validation approach used independent null simulations with the same gene length as *gtsB* under a simpler evolutionary model, in which purifying selection is uniformly strong at nonsynonymous sites while synonymous sites evolve neutrally. Across 39 null replicates, no 4-fold synonymous site was ever found clustering with neighboring nonsynonymous sites, whereas 8% met this criterion in *gtsB*, indicating that this pattern is unlikely to arise by chance. The first two PCs also explained a higher proportion of variance in *gtsB*'s patterns of substitution at neighboring nonsynonymous and 4-fold synonymous sites (75%, Fig. 2) than in null replicates (60.86% to 66.63%, Table S15). A similar value was observed for *gtsD* (67%, Fig. 2); however, since *gtsD* is slightly larger than *gtsB* and no null independent simulation matching its length was generated, this result should be considered suggestive rather than confirmatory. Together, these findings suggest that purifying selection at 4-fold synonymous sites varies in strength, potentially reflecting additional structural or functional constraints in regions influencing gene expression, protein structure, or molecular interactions (Hunt et al. 2014; Ooi et al. 2022).

It is also worth noting that our approach does not constitute a formal statistical test for selection; rather, it relies on the converging support of multiple validation lines of evidence as described above. Beyond these findings, our independent null was built on several simplifying assumptions such as a single uniform *ω* across nonsynonymous sites, fixed branch lengths, and no rate heterogeneity. Further work incorporating more realistic evolutionary models is needed to better quantify the relative contributions of mutation bias and selection in shaping substitution patterns at 4-fold synonymous sites.

More broadly, estimates of evolutionary constraint are influenced by the direction of selection in relation to mutational biases and may vary over time (Lawrie et al. 2011, 2013). For instance, a site that switches between two distinct states depending on the environment may exhibit increased substitution patterns and greater polymorphism, despite being functionally important and constrained by selection. Given this asymmetry, there may be other constrained sites in the sequences that we are unable to detect, as is the case of some sites in cluster 2 of *gtsB* in Fig. 2a that have been shown experimentally to have beneficial effects (Bailey et al. 2014; Lebeuf-Taylor et al. 2019).

On the other hand, our comparative approach did recover 4-fold synonymous sites from codons 10 and 38 in *gtsB*, codons where, in a previous study, highly beneficial mutations emerged spontaneously in populations of *P. fluorescens* evolving under low glucose conditions (Bailey et al. 2014). This finding provides additional evidence that these codons are broadly constrained and may play a role in adaptation beyond the specific conditions used in the experiment. It is important to note that the constrained substitution patterns observed at candidate 4-fold synonymous sites across the phylogeny does not exclude specific changes at those sites being beneficial in particular ecological contexts. Mutations with deleterious effects across most environments can have beneficial effects in others, a phenomenon known as *conditionally beneficial mutations*, where the fitness effect of a mutation depends on the genetic and environmental context (Elena and De Visser 2003; Weinreich et al. 2005). The phylogenetic signal of constraint and the experimental evidence for fitness effects at two constrained synonymous sites in *gtsB* are therefore not contradictory but reflect the same sites being subject to different selective pressures depending on the genetic background and environmental conditions. More experimental research is needed to quantify the fitness effects of the other six candidate synonymous sites under strong purifying selection in *gtsB* as well as the ten sites identified in *gtsD* and to potentially identify the underlying cause(s) behind these patterns.

It is notable that, among the many mechanistic explanations for fitness effects of synonymous mutations, none that we tested emerged as a single, general explanation for the patterns of substitution we recover in our study. Whether such a general mechanism exists or whether the fitness effects of synonymous mutations are a by-product of other factors governing the evolution of local sequence motifs is a major empirical challenge. It could be, for example, that epistatic interactions and local/global differences in genomic context, factors we have not considered here, contribute to the propensity of a synonymous site to exhibit non-neutral fitness. An expanded study including a more phylogenetically diverse sample could shed light on some of these issues.

This work, although limited to two genes, contributes to the increasing body of evidence that synonymous sites are not uniformly neutral and can, in other genomic contexts, contribute to adaptation. We identify distinct classes of synonymous sites likely resulting from variation in selection, suggesting it may be possible to identify synonymous variants under strong selection by analyzing their substitution rate and profile. This may be particularly effective in species with large population sizes, like bacteria, where even mutations with small fitness effects should be impacted by natural selection (Rocha 2018). Bayesian stochastic character mappings combined with HCPC and consensus clustering provide a robust framework for studying selective constraints across genes and identifying candidate synonymous sites under strong purifying selection. These candidates could then be empirically tested to determine the magnitude of their fitness effects and their role in adaptation across different environments and conditions.

## Materials and Methods

The methods used are described in detail below, with the main steps summarized in Fig. S17.

### Data and Tree Construction

A maximum likelihood phylogenetic tree (Fig. S1b) was inferred using a protein sequence alignment of 100 single-copy orthologous genes and the same parameters as (Hesse et al. 2018) for 258 Pseudomonas strains. *Vibrio vulnificus* CMCP6 was used as an outgroup, a consensus tree as generated using IQ-TREE (version 2.0.7) (Nguyen et al. 2015) using the LG4M model (Four-matrix model fused with Gamma rate heterogeneity) (Le et al. 2012). Branch supports were assessed using the combination of 1,000 nonparametric bootstrap and 1,000 aLRT (Guindon et al. 2010).

Ortholog DNA sequences of *gtsB* (*n* = 259 species) and *gtsD* (*n* = 255 species, orthologous sequences for four species were not available) in the phylogeny were obtained by BLASTn searches of each genome's coding sequences against the reference gene sequence of *P. fluorescens SBW25*, using an *e*-value threshold of 1e−5 from NCBI's BLAST (Sayers et al. 2022) and JGI BLAST (Chen et al. 2017). Only genomes with a single significant hit were retained to ensure unambiguous ortholog assignment. Ortholog identity was further confirmed by gene annotation where available. Ortholog sequences were subsequently aligned with MAFFT (version 7.487) (Katoh and Standley 2013) and trimmed with trimAl 1.4 (Capella-Gutiérrez et al. 2009) sites for which more than 90% of taxa contained a gap, leading to a final dataset with 909 positions in *gtsB* (the same length as *P. fluorescens* SBW25) and for *gtsD* 1,131 positions (in *P. fluorescens* SBW25 this gene has a length of 1,161 nucleotides, but 25 nucleotides from positions 1,106 to 1,130 are absent in the majority of the analyzed strains).

Phylogenetic tree visualization and annotation was performed in the interactive tree of life web portal (Letunic and Bork 2021).

### Phylogenetic Reconstruction

Phylogenetic analyses of *gtsB* and *gtsD* alignments were run independently and were performed under the CAT-GTR (general time reversible) model (Tavare 1986; Lartillot and Philippe 2004) with a four-category discretized gamma distribution (Γ) to accommodate rate heterogeneity among sites (Yang 1994) using PhyloBayes-MPI (version 1.8c; Lartillot et al. 2013). Though rarely utilized at the nucleotide level, a recent study shows that this model outranks all other commonly used nucleotide substitution models (Bujaki et al. 2023).

All prior distribution settings for branch lengths and for the CAT-GTR-Γ model were the PhyloBayes-MPI default values, and as a fix parameter, we used the topology of the phylogenetic tree reconstructed in the previous step. Each analysis involved running at least two independent chains for at least 36,000 generations. Convergence and the appropriate burn-in proportion were first assessed visualizing the summary statistics recorded in the trace files as a function of the number of iterations using the Tracer program (Rambaut et al. 2018). Convergence between chains was then formally confirmed using the tracecomp program in PhyloBayes-MPI, after discarding the determined burn-in proportion (∼10%).

### Posterior Mappings and Posterior Predictive Mappings

For each gene sequence, we sampled posterior mappings and posterior predictive mappings across 1,000 evenly spaced generations (parameter space configurations) from the posterior distribution of one of the converged chains obtained in the previous step in PhyloBayes-MPI, using the CAT-GTR-Γ model as for the original chain. As a result, we obtained 1,100 posterior mappings and 1,100 posterior predictive mappings for *gtsB* and 979 posterior mappings and 979 posterior predictive mappings for *gtsD*.

### Posterior Predictive *P*-values

We assessed the adequacy of the molecular model of evolution by calculating the substitution rate, defined as the mean number of substitutions, derived from the posterior mappings sampled for each gene and position. Similarly, we computed the substitution rate from the posterior predictive mappings using the same procedure.

Posterior predictive *P*-values (PP) were computed from the lower and upper tails of the posterior predictive distribution as the proportion of draws in which the substitution rate calculated from the posterior predictive mappings either exceeded (upper tail) or fell below (lower tail) the substitution rate derived from the observed posterior mappings, following (Bollback 2002; Table S1).

Since PPs are not uniformly distributed under adequate model fit but instead concentrate around intermediate values near 0.5 (Fabreti et al. 2024), they were not evaluated against a fixed significance threshold. Following Fabreti et al. (2024), PP values deviating toward 1 on the upper tail or toward 0 on the lower tail indicate that the observed substitution mapping consistently exceeded the posterior predictive mappings across all draws, while the opposite pattern, PP values toward 0 on the upper tail or toward 1 on the lower tail, indicates that the posterior predictive mappings consistently exceeded those of the observed data. In both cases, such deviations from 0.5 reflect a discrepancy between observed and posterior predictive substitution maps. Model adequacy was therefore assessed by examining how closely PP values approached 0.5 on both tails, complemented by effect size metrics. To assess the magnitude of model deviation, we calculated *Z*-scores, as the difference between the observed and posterior predictive substitution rates, normalized by the standard deviation of the posterior predictive distribution, and PPES (Doyle et al. 2015; Fabreti et al. 2024), calculated as the absolute difference between the observed substitution rate and the median of the posterior substitution rate, normalized by the standard deviation of the posterior predictive distribution.

### Analysis of Associated Biological Variables

The CAIcal server (Puigbò et al. 2008) was used to determine codon adaptation weights (*w*) for at least one species from each of the 12 Pseudomonas groups and each of the 10 *P. fluorescens* subgroups in the phylogeny (*n* = 30, Table S17). For each species included, the synonymous codon frequencies used as the reference table were obtained from the Codon and Codon Pair Usage Tables (CoCoPUTs) (Alexaki et al. 2019), which are calculated across all genes in a genome, displaying genome-wide codon usage for each species. For each group of synonymous codons, a weight of 1 (*w* = 1) indicates the most frequently used codon in a synonymous group, whereas values between 0 and 1 represent the usage frequency of all other synonymous codons in the same group relative to the most frequent one. We next calculated the average, standard deviation, and coefficient of variation (CV) for each codon weight (W) across the examined species (Table S17). We then used the obtained mean values as supplementary variables in the HCPC analysis. We observed substantial variability (0.3 < CV < 0.73) at eight codons from amino acids with 4-fold synonymous sites (valine, proline, threonine, glycine, and alanine) ending predominantly in A and occasionally in T, contrasting with the general preference for C or G-ending codons at third positions in Pseudomonas.

The RSCU for each gene alignment was computed using DAMBE (version 7) (Xia 2018). According to Sharp and Li (1986a, 1986b), RSCU is the product of the observed frequency of a codon and the expected frequency for any given amino acid. A synonymous codon with a higher frequency of occurrence has an RSCU > 1; an RSCU = 1 for equally used synonymous codons, whereas one with a lower frequency has an RSCU < 1. As before, we computed the average, standard deviation, and CV of the RSCU for each position across the examined species (Table S18). The resulting mean values were then used as supplementary variables in the HCPC analysis. RSCU values were highly conserved across species, with all CVs below 0.01.

### Observed Codon-ending Relative Probabilities Calculation

For each codon along *gtsB* and *gtsD*, substitution profiles (the 12 transition probabilities) at the first, second, and third positions inside the codon were assumed to be independent and used to estimate the probability of every possible codon as the product of the corresponding position-specific probabilities. Then, all probability combinations resulting in the same codon were summed, generating a probability distribution across all 64 codons. From this distribution, we retained the four synonymous codons at positions encoding valine, glycine, alanine, proline, or threonine, based on the corresponding reference gene sequence of *P. fluorescens SBW25*. Because these synonymous codons vary only at the third position, their probabilities were normalized to yield a relative probability for each of the four codon endings (A, G, C, T). These relative probabilities were included as active variables to characterize relationships between substitution rate, codon usage bias, and amino acid identity.

### Protein Topological Organization

To examine if there was any relationship between a protein structure and the patterns of substitutions at each position in a protein sequence, we used for the *gtsB* gene protein topology prediction DeepTMHMM (version 1.0.8) (Hallgren et al. 2022). For the *gtsD* gene, we used PSIPRED (version 4.0) (Buchan and Jones 2019). As explained above, the protein topological organization, in *gtsB* corresponds to the most probable location and orientation of transmembrane helices in the sequence (cytoplasm, membrane, and periplasm), while in *gtsD* corresponds to different types of secondary structures that contribute to the overall 3D shape of the protein (strand, helix, coil).

### Multidimensional Analysis and Initial Cluster Evaluation

HCPC is a technique that combines PC analysis (PCA) for reducing the number of dimensions and hierarchical clustering to achieve reliable grouping by eliminating noise (Husson et al. 2017).

The variables were standardized for the PCA; in all cases, we considered the first two PCs. The Ward method with Euclidean distance was then used to perform the hierarchical clustering, represented with a dendrogram. We used the dendrograms and Davies–Bouldin index (Davies and Bouldin 1979) to initially evaluate the number of clusters that better accounted for the data.

We used the *η*^2^ statistic, as implemented in FactoMineR (Lê et al. 2008), to quantify the proportion of total variance in each quantitative variable explained by cluster membership. Values close to 1 indicate strong discrimination between clusters, whereas values near 0 indicate little to no association. Associations between cluster membership and qualitative supplementary variables were assessed using *χ*^2^ tests, and over- or underrepresented categories within clusters were identified using the V-test statistic implemented in FactoMineR (Lê et al. 2008).

All analyses were conducted in R (version 4.3.1, http://www.r-project.org) using the libraries FactoShiny (Husson et al. 2010) and FactoMineR (Lê et al. 2008) and reproduced in Python 3.12.4 for graphical purposes.

### Cluster Stability and Determination of the Optimal Number of Clusters (*K*)

For each gene, cluster stability was evaluated using consensus clustering (Monti et al. 2003) in R. For each candidate number of clusters (*K* = 2 to 7), 5,000 subsamples were generated without replacement, retaining 80% of sites per iteration. When analyses included neighboring nonsynonymous sites and 4-fold synonymous positions, subsampling was stratified to ensure equal representation of both site types within each iteration. Robustness was further assessed by repeating the analysis using subsampling fractions of 0.7 and 0.9.

As in the full dataset, within each subsample, sites were projected onto the first two PCs and clustered using Ward's hierarchical method implemented in FactoMineR. For each *K*, a consensus matrix (*M*) was constructed summarizing the proportion of subsampling iterations in which pairs of sites were assigned to the same cluster. Final cluster assignments were obtained by hierarchical clustering of the consensus dissimilarity matrix (1 − *M*).

Cluster stability was evaluated using multiple complementary metrics, including the empirical CDF of consensus values, the change in CDF area across *K* (Δ*K*), the proportion of ambiguous clustering (PAC; Şenbabaoǧlu et al. 2014), mean within-cluster consensus, and site-level stability.

The Δ(*K*) curve was examined to identify points beyond which additional clusters produced diminishing changes in consensus structure (“elbow” criterion; Monti et al. 2003). Because Δ(*K*) can be influenced by large structural splits at small *K*, it was interpreted jointly with other stability metrics rather than selecting the maximum Δ(*K*) automatically as suggested by Şenbabaoǧlu et al. (2014).

Lower PAC values and higher within-cluster consensus indicate more stable partitions. The optimal number of clusters was selected based on agreement among these metrics, robustness across subsampling fractions, and biological interpretability.

Results were summarized using consensus matrix heatmaps, CDF curves, Δ*K* plots, and site-level stability distributions (Figs. S3, S5, S8, and S10), and all stability metrics are reported in Tables S4 and S9.

### Entropy Analysis

We calculated Shannon entropy (Shannon 1948) based on the relative frequencies of the 12 possible nucleotide substitutions inferred from posterior mappings. For each site, substitution counts were first averaged across mappings and converted to proportions by dividing each substitution count by the total substitution count at that site. Shannon entropy was then computed as:


$$H=-\overset{12}{\underset{k=1}{\sum }}\;p_{k}\mathrm{lo}g_{2}(\;p_{k})$$

where *p_k_* is the proportion of substitutions of type *K*. Categories with zero counts were assigned a contribution of zero to the entropy calculation.

Entropy values were normalized by dividing by the maximum possible entropy (log_2_ 12), resulting in values ranging from 0 to 1. Differences in normalized entropy among site classes within each gene (constrained 4-fold synonymous sites, less constrained synonymous sites, and neighboring nonsynonymous sites) were evaluated using linear models. Because variance differed among site classes, inference was based on heteroscedasticity-robust standard errors (HC3). Pairwise comparisons were performed using estimated marginal means with Holm adjustment for multiple testing.

### Posterior Predictive Permutations

For each posterior predictive mapping generated under CAT + GTR+Γ4 for *gtsB* (*N* = 1,100) and *gtsD* (*N* = 976) (Table S1), respectively, substitution rates and profiles were extracted from nonsynonymous neighboring sites and 4-fold synonymous sites, pooled together, and then shuffled while keeping each site's rate and profile paired. This permutation preserves the overall rate distribution while breaking any association between substitution pattern and codon position, generating a null distribution under the hypothesis that second and third codon positions are exchangeable. Then to each permuted mapping, the HCPC approach was applied using the same parameters as the observed analysis, the first two PCs and the optimal number of clusters determined by consensus clustering (*K* = 4 and *K* = 5 for *gtsB* and *gtsD*, respectively), with the cluster containing the sites with the lowest mean substitution rate (cluster 1) identified as the constrained cluster. The number of 4-fold synonymous positions assigned to the constrained cluster was recorded for each mapping, generating an empirical null distribution of constrained synonymous sites.

Three metrics were calculated to assess significance relative to this null distribution. First, a gene-level empirical *P*-value assessed whether the total number of constrained 4-fold synonymous sites in the real data is smaller than expected by chance. Second, a *Z*-score provided an effect size measure of departure from null expectations. Finally, a site-level empirical *P*-value was used to assess whether each specific candidate site is consistently assigned to the constrained cluster beyond chance, with values near or equal to 0.001, after applying the +1 correction of Phipson and Smyth (2010) to avoid *P*-values equal to zero, indicating that permuted mappings rarely assigned that site to the constrained cluster by chance.

### Independent Replicate Simulations

We used AliSim (Ly-Trong et al. 2022, 2023) implemented in IQ-TREE v3.0.1 to generate 40 simulated coding-sequence alignments with the same length as *gtsB* (*n* = 909 nucleotide positions). Simulations were conducted on the phylogenetic topology inferred from the 100 core genes in 258 Pseudomonas species, with branch lengths fixed at 0.05. We used the Muse and Gaut (1994) (MG94) codon substitution model, combined with the F3 × 4 frequency model (Muse and Gaut 1994), which predicts codon frequencies from the empirical nucleotide frequencies in all three codon positions, with a transition/transversion bias *κ* (=3) and the *dN*/*dS* ratio *ω* (=0.02).

For each simulated dataset, we conducted a phylogenetic analysis in PhyloBayes under the same conditions used for the observed data, running chains until at least 36,000 generations. One replicate failed to converge and was excluded, leaving a total of 39 independent replicates. After discarding burn-in, we generated posterior substitution mappings from the remaining samples, yielding ∼1,000 mappings per replicate for downstream analysis.

For each simulated alignment, a reference sequence was determined by identifying the most frequent codon across all species at every codon position, and the corresponding amino acid was assigned using the standard genetic code. In cases where multiple codons were equally frequent at a site, the consensus codon was assigned alphabetically. Four-fold synonymous sites along with their neighboring nonsynonymous second-position sites were kept in each replicate and used for downstream analysis.

### TSS Prediction

The prediction of internal promoters in the *gtsB* (*n* = 259 Pseudomonas species) and *gtsD* (*n* = 255 Pseudomonas species) genes was carried out using SAPPHIRE. CNN (Coppens et al. 2022) .We took as reference the promoters predicted for *P. fluorescens SBW25* using a *P* < 0.05 threshold across the species present in each gene alignment.

### mRNA Folding Stability

We used mfold (Zuker 2003) to predict and compare mRNA structure and stability effect of each of the 4-fold alleles at identified constrained sites in *gtsB* and *gtsD* gene sequences of *P. fluorescens SBW25*.

## Supplementary Material

evag241_Supplementary_Data

## Data Availability

The data underlying this article are available in the article in its online Supplementary material. Gene DNA alignments, phylogenetic consensus tree and code are available in the GitHub repository: https://github.com/angelaalonsom30/supplementary_data_synonymous_mutations_under_selection.
