# Supplementary material for: Identifying Patterns of Selection at Synonymous Sites in a Pseudomonas Phylogeny
Source: Genome Biol Evol. 2026 Sep 29;18(10):evag241. doi: 10.1093/gbe/evag241 (PMC13689711; doi:10.1093/gbe/evag241)
Supplement: Supplementary file 1 [file evag241_supplementary_data.zip › Supplementary_Figures_GBE-250330-R3_2.pdf]

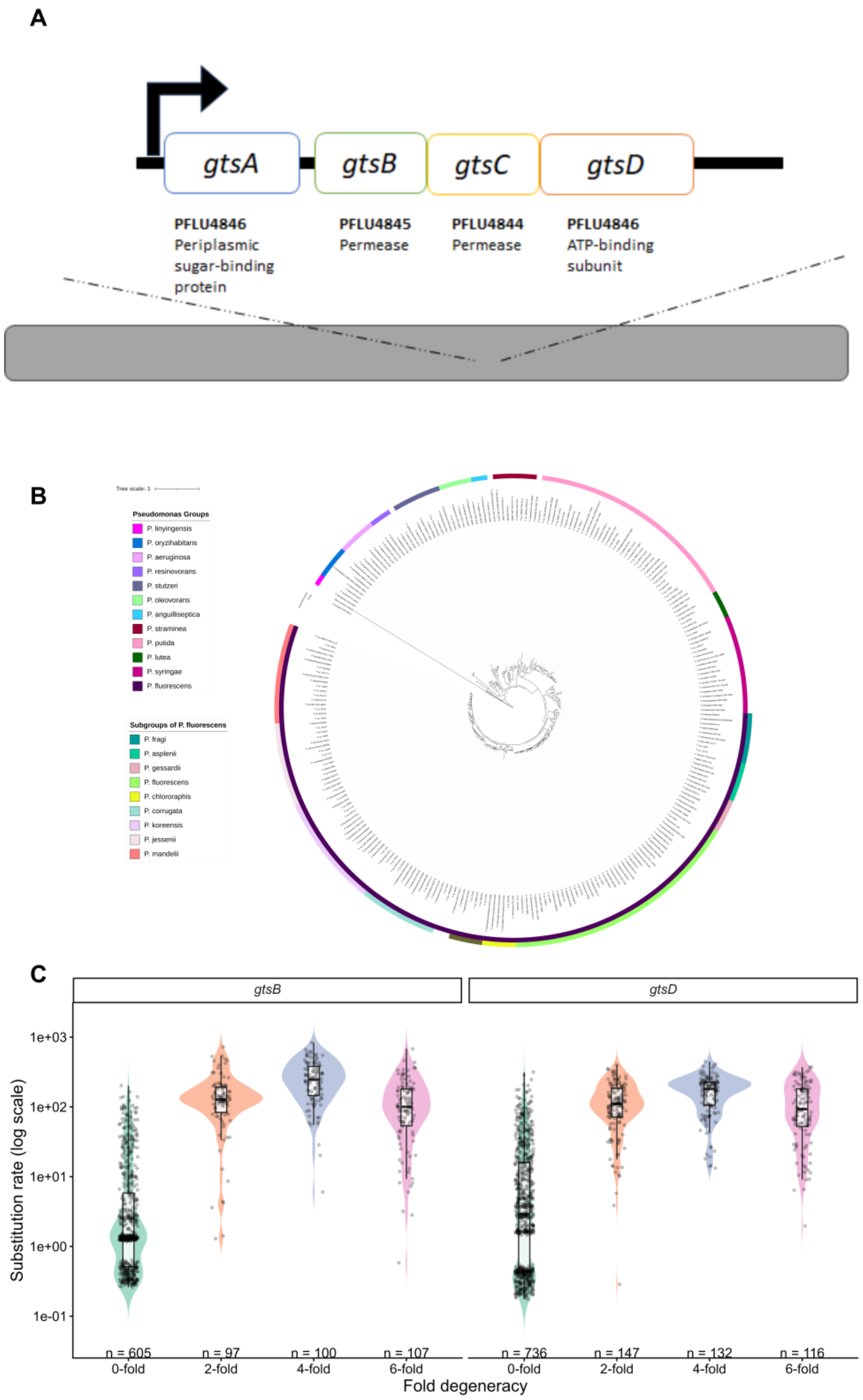

Figure S1. Organization of the *gtsABCD* operon and phylogenetic tree of Pseudomonas species. (A) Gene architecture of the *gtsABCD* operon. (B) Maximum likelihood phylogeny based on protein sequence alignments of 100 single-copy orthologous genes from 258 strains of Pseudomonas species. (C) Substitution rates distribution by fold degeneracy in *gtsB* and *gtsD*.

A

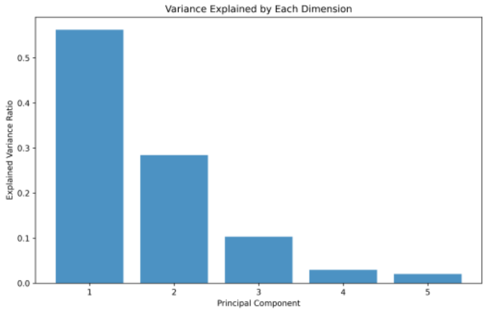

B

PC	Eigenvalues	Explained Variance Ratio	Cumulative Explained Variance
1.0	3.9774671504063557	0.5625274969860415	0.5625274969860415
2.0	2.009968208790797	0.2842669323861269	0.8467944293721684
3.0	0.729322835624484	0.10314708675260555	0.949941516124774
4.0	0.20973162446245716	0.02966204403111893	0.9796035601558929
5.0	0.14421725142297925	0.020396439844107057	1.0

C

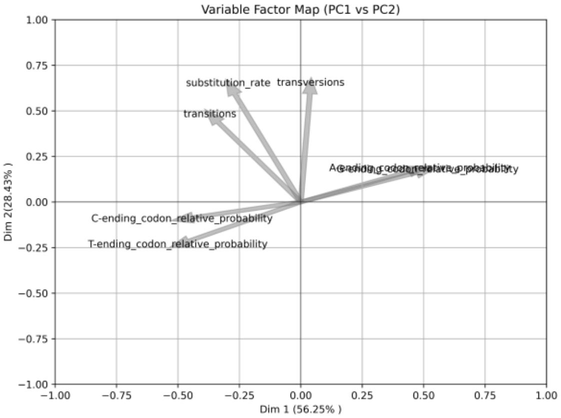

D

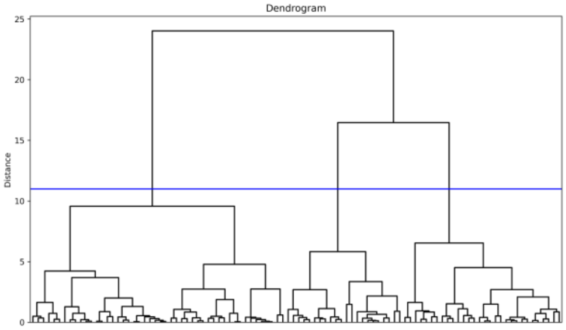

E

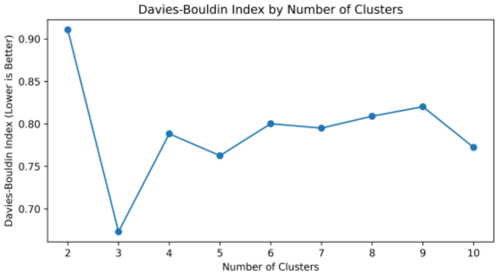

Figure S2. Hierarchical Clustering on Principal Components metrics on four-fold synonymous sites on the *gtsB* gene (n=100) using seven quantitative variables. (A) Proportion of Variance plot. (B) Proportion of Variance values. (C) Variable factor map on PC1 vs PC2. (D) Hierarchical clustering dendrogram using the PCA's first two dimensions. The height of the branches indicates the dissimilarity between clusters. The blue line cuts the tree into four clusters. (E) Davies–Bouldin index by number of clusters.

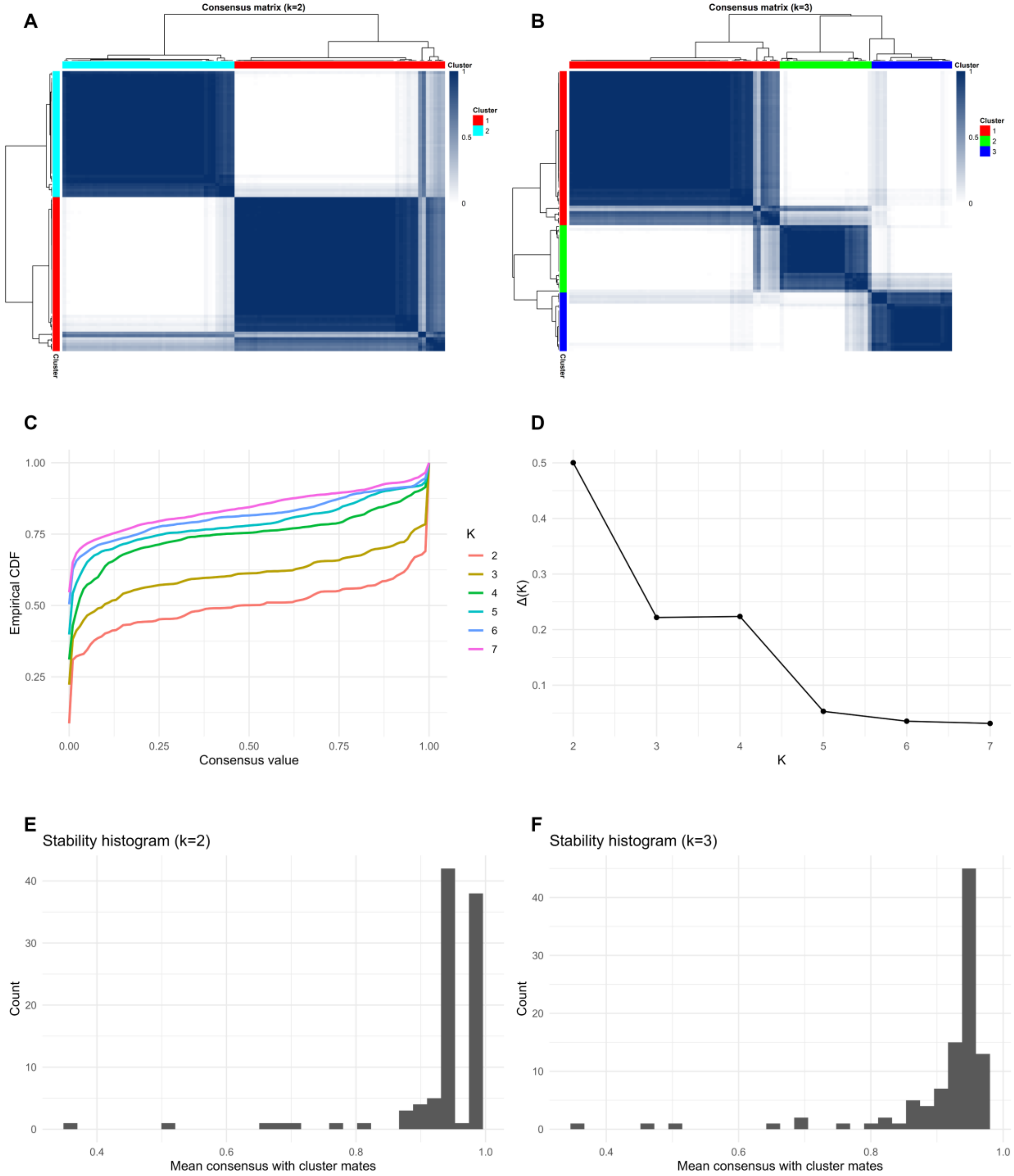

Figure S3. Consensus clustering diagnostic plots on the four-fold synonymous sites in the *gtsB* gene (n = 100). Results are from a random 80% subset of the total number of four-fold synonymous sites in the gene. Plots are display for K = 2 and K =3 as both have are supported by consensus clustering (Table S3). (A-B) Consensus matrix heatmaps. (C) CDF curve. (D) ΔK plot. (E-F) Site-level stability distributions. K = 3 was selected as it retained high within-cluster consensus with only a modest reduction in stability, indicating additional consistent structure.

A

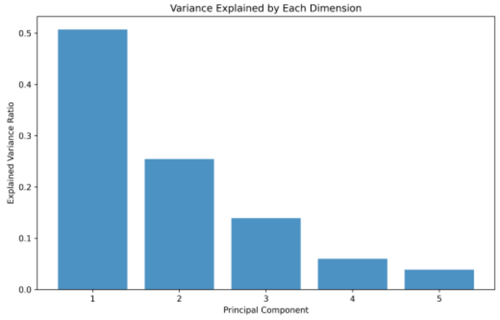

B

PC	Eigenvalues	Explained Variance Ratio	Cumulative Explained Variance
1.0	3.5773961668077825	0.5071849543850865	0.5071849543850865
2.0	1.7960475627925916	0.254634448837478	0.7618194032225645
3.0	0.9828416542588896	0.13934226916440978	0.9011616723869742
4.0	0.4230347485551929	0.059975705693431074	0.9611373780804052
5.0	0.2741149820893544	0.03886262191959465	0.9999999999999999

C

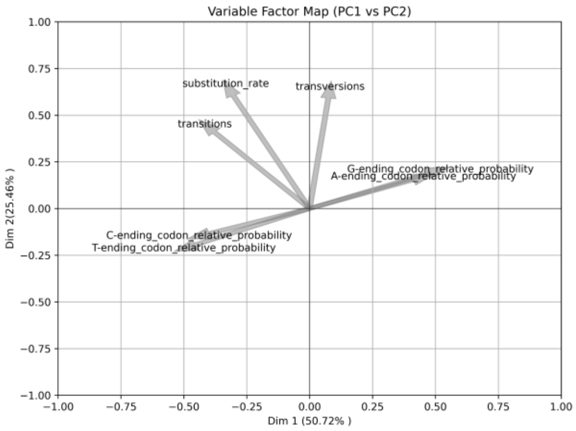

D

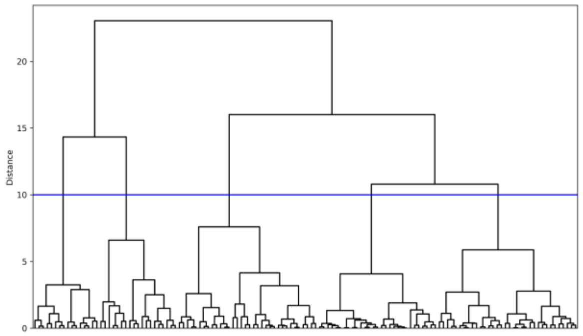

E

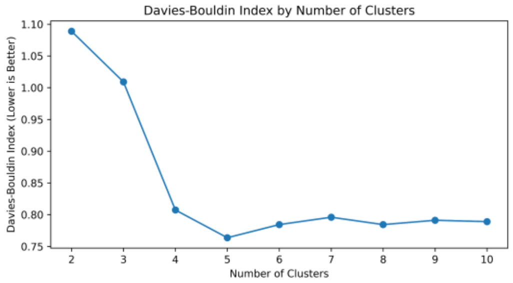

Figure S4. Hierarchical Clustering on Principal Components metrics on four-fold synonymous sites on the *gts*D gene (n=132) using seven quantitative variables. (A) Proportion of Variance plot. (B) Proportion of Variance values. (C) Variable factor map on PC1 vs PC2. (D) Hierarchical clustering dendrogram using the PCA's first two dimensions. The height of the branches indicates the dissimilarity between clusters. The blue line cuts the tree into five clusters. (E) Davies–Bouldin index by number of clusters.

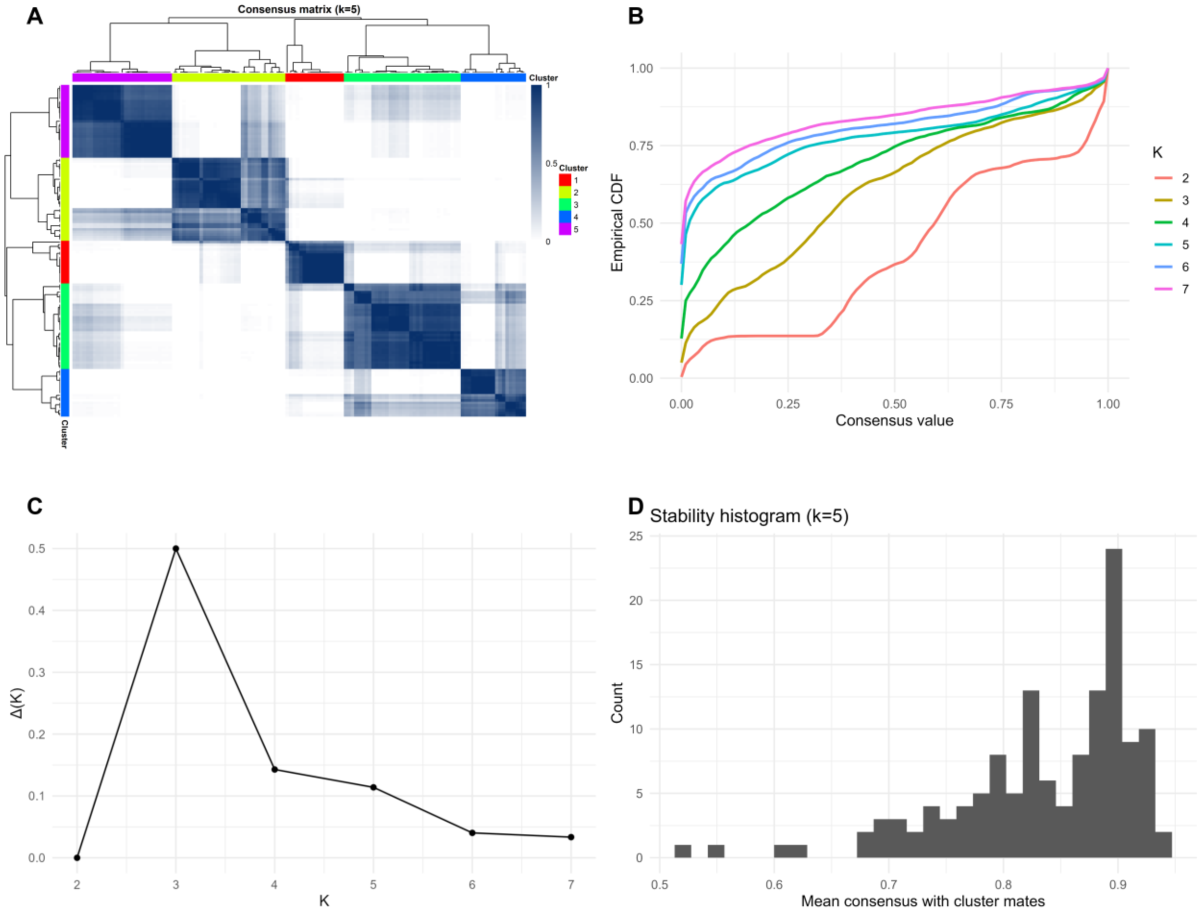

Figure S5. Consensus clustering diagnostic plots on the four-fold synonymous sites in the *gtsD* gene (n = 132). Results are from a random 80% subset of the total number of four-fold synonymous sites in the gene. Plots are display for K = 5 as this cluster number is supported by consensus clustering (Table S3). (A) Consensus matrix heatmap. (B) CDF curve. (C) ΔK plot. (D) Site-level stability distribution.

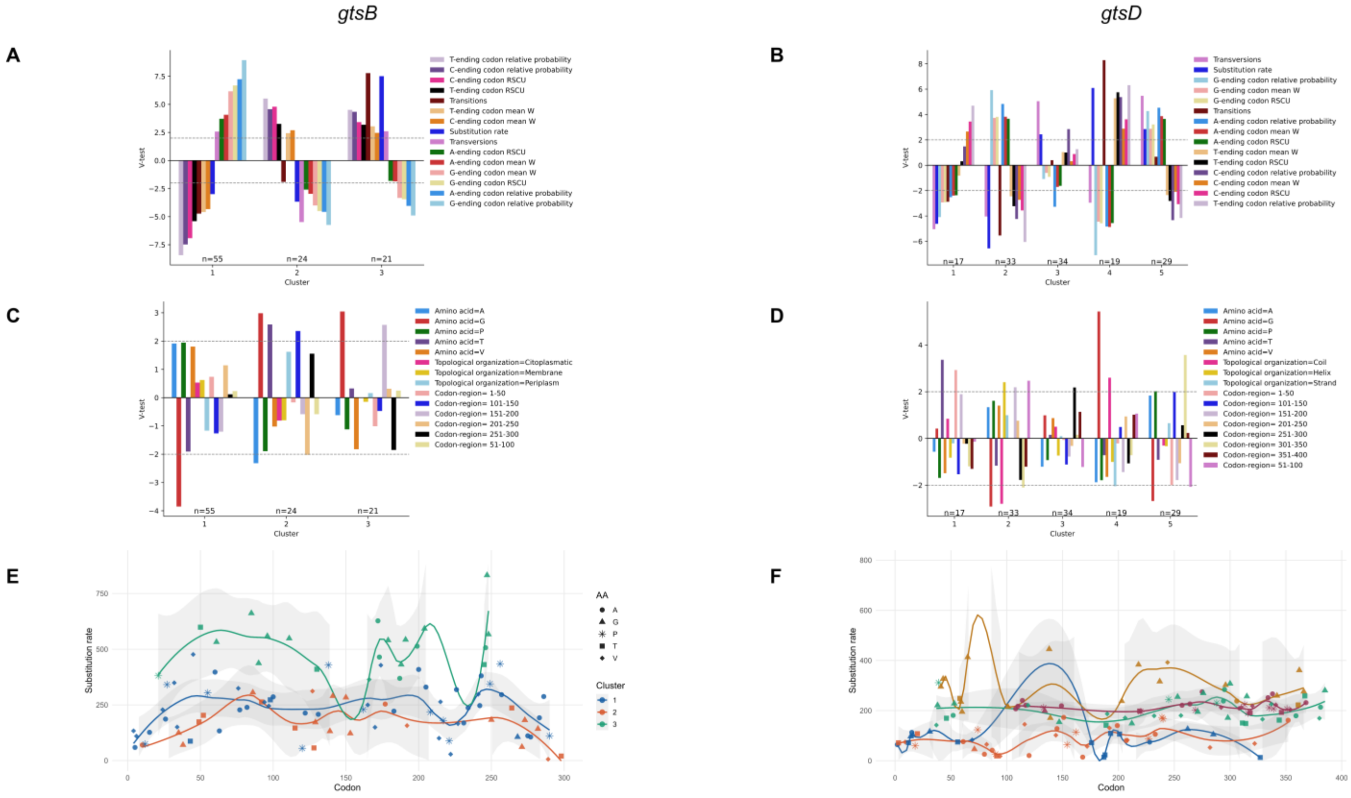

Figure S6. Relationship between active and supplementary quantitative (codon usage metrics) and qualitative variables by clusters on four-fold synonymous sites of *gtsB* (panels A and C, n = 100) and *gtsD* (panels B and D, n = 132). Results derive from a random 80% subset of the total number of four-fold synonymous sites in the gene. Plots are displayed for K = 3 (*gtsB*) and K = 5 (*gtsD*), respectively. The V-test represents a standardized deviation between the average of each variable among positions in a cluster compared to the general average. Horizontal dotted lines indicate a cutline, where V-test scores equal to or greater than 2 or equal to or less than -2 are considered significant ($P < 0.05$). (E and F) Substitution rate at four-fold synonymous sites along gene codon positions. Points represent individual sites. Solid lines represent LOESS smoothing (span = 0.35). Color indicates cluster; shape indicates amino acid.

A

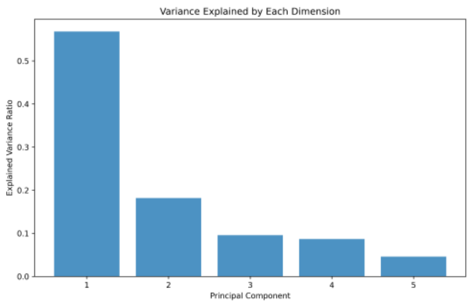

B

PC	Eigenvalues	Explained Variance Ratio	Cumulative Explained Variance
1.0	8.56473751352183	0.5681275883969482	0.5681275883969482
2.0	2.7422587774120197	0.1819031655683307	0.7500307539652789
3.0	1.442377247752014	0.09567769076755028	0.8457084447328291
4.0	1.308646839659826	0.08680690703076849	0.9325153517635976
5.0	0.6907968457582947	0.04582285743530022	0.9783382091988978

C

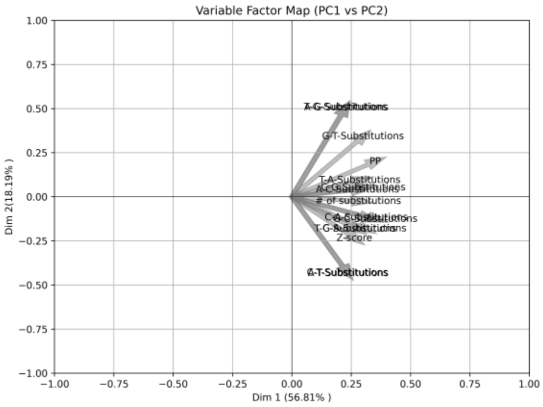

D

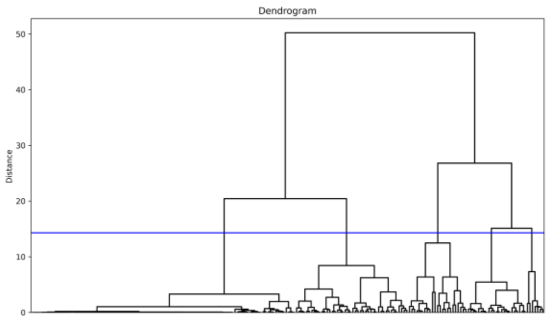

E

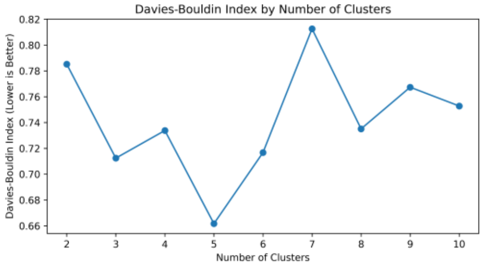

Figure S7. Hierarchical Clustering on Principal Components metrics on the second neighboring and four-fold synonymous sites in *gtsB* (n = 200) using 15 quantitative variables. (A) Proportion of Variance plot. (B) Proportion of Variance values. (C) Variable factor map on PC1 vs PC2. (D) Hierarchical clustering dendrogram using the PCA's first two dimensions. The height of the branches indicates the dissimilarity between clusters. The blue line cuts the tree into five clusters. (E) Davies–Bouldin index by number of clusters.

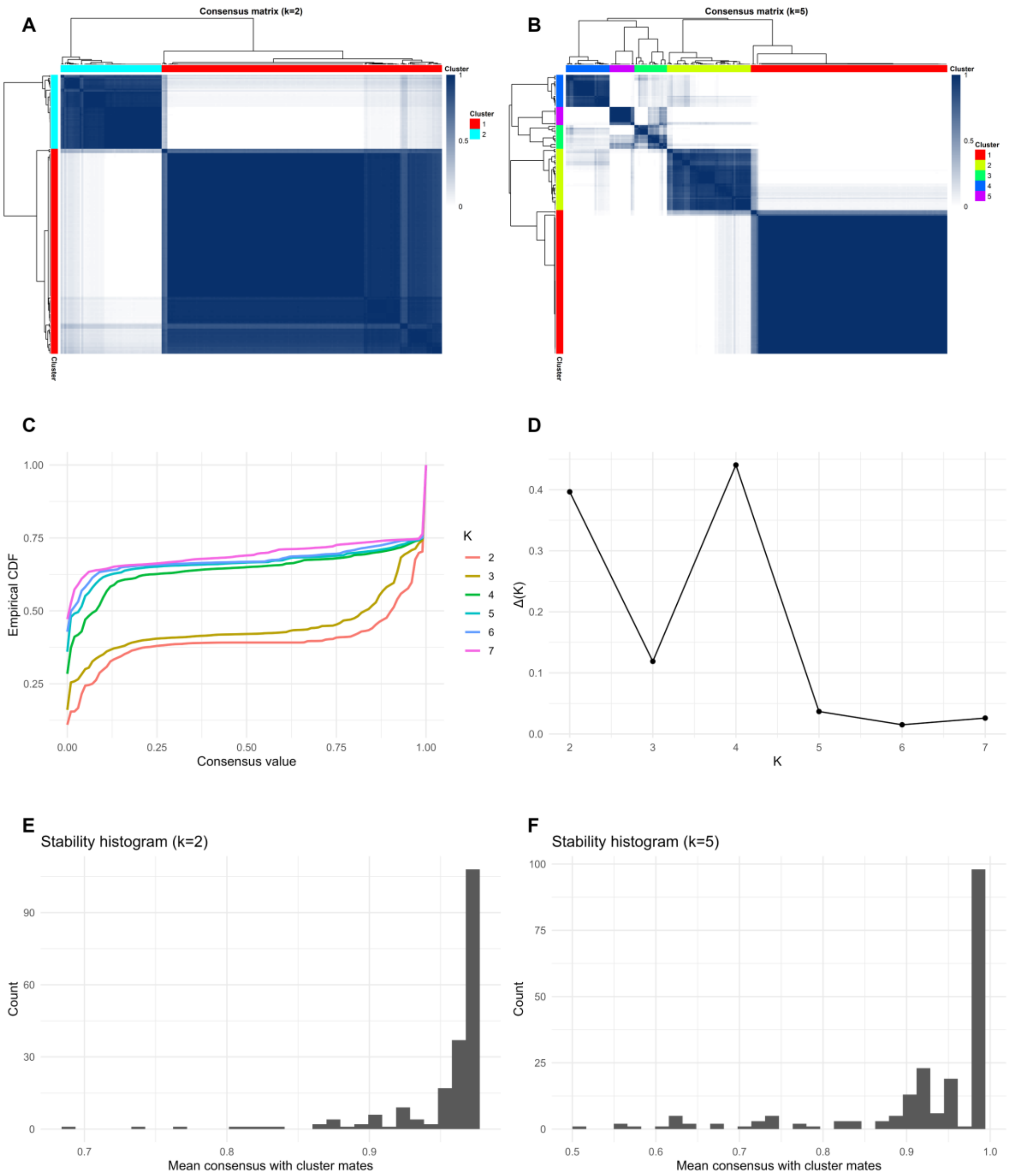

Figure S8. Consensus clustering diagnostic plots for neighboring second positions and four-fold synonymous sites in *gtsB* (n = 200). Results are shown for a random 80% subsample of the total number of four-fold synonymous sites and second positions within the gene; subsampling of each site type was performed independently. Plots are displayed for K = 2 and K = 5, as both values were supported by consensus clustering (Table S8). (A–B) Consensus matrix heatmaps. (C) CDF curve. (D) ΔK plot. (E–F) Site-level stability distributions. K = 5 was selected because additional structure became apparent at K = 4 (ΔK = 0.44), and assignment ambiguity was markedly reduced at K = 5 with only a modest reduction in stability, indicating consistent additional structure.

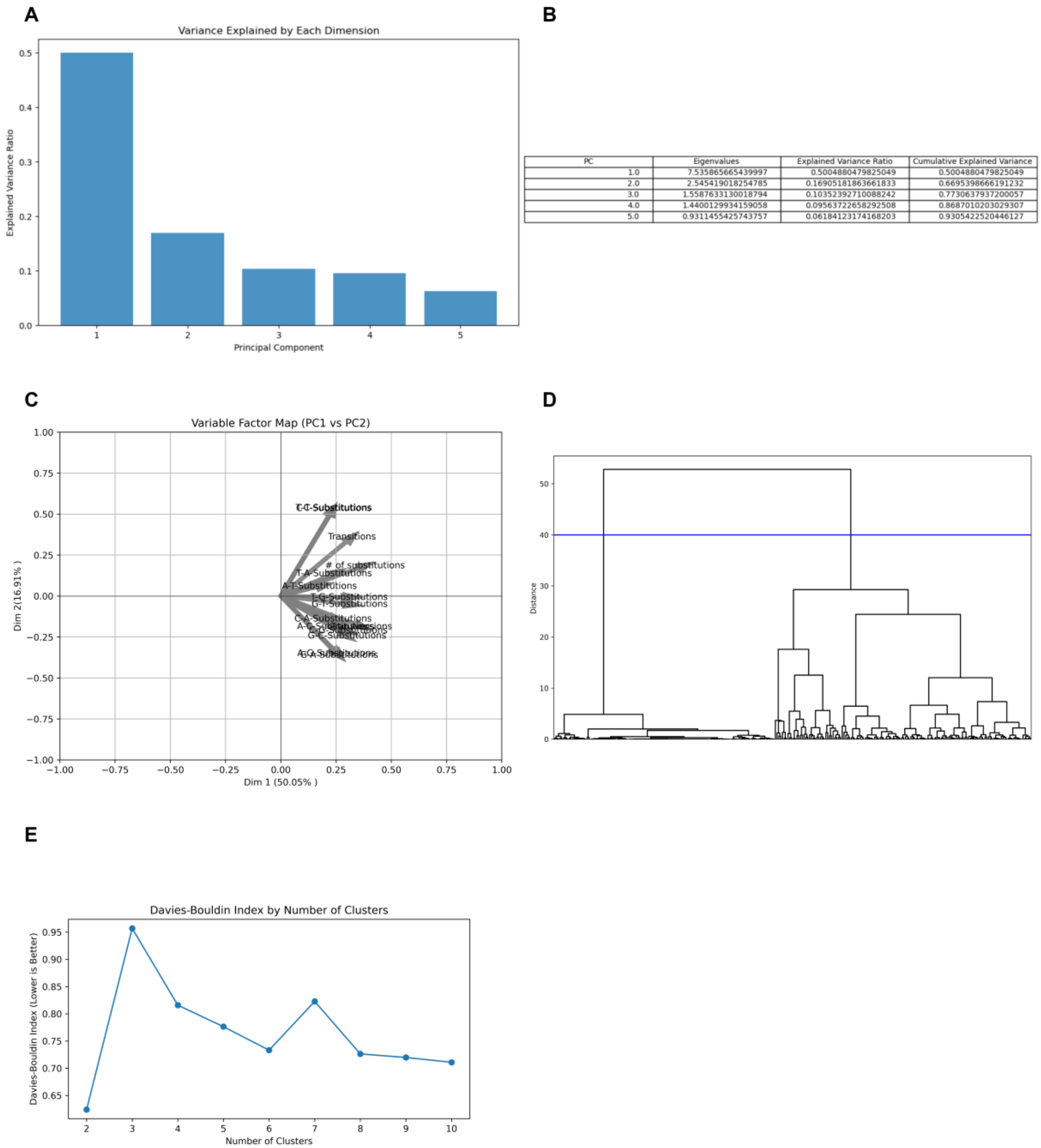

PC	Eigenvalues	Explained Variance Ratio	Cumulative Explained Variance
1.0	7.535865665439997	0.5004880479825049	0.5004880479825049
2.0	2.545419018254785	0.16905181863661833	0.6695398666191232
3.0	1.5587633130018794	0.10352392710088242	0.7730637937200057
4.0	1.4400129934159058	0.09563722658292508	0.8687010203029307
5.0	0.9311455425743757	0.06184123174168203	0.9305422520446127

Figure S9. Hierarchical Clustering on Principal Components metrics on the second neighboring and four-fold synonymous sites in *gtsD* (n = 264) using 15 quantitative variables. (A) Proportion of Variance plot. (B) Proportion of Variance values. (C) Variable factor map on PC1 vs PC2. (D) Hierarchical clustering dendrogram using the PCA's first two dimensions. The height of the branches indicates the dissimilarity between clusters. The blue line cuts the tree into two clusters. (E) Davies–Bouldin index by number of clusters.

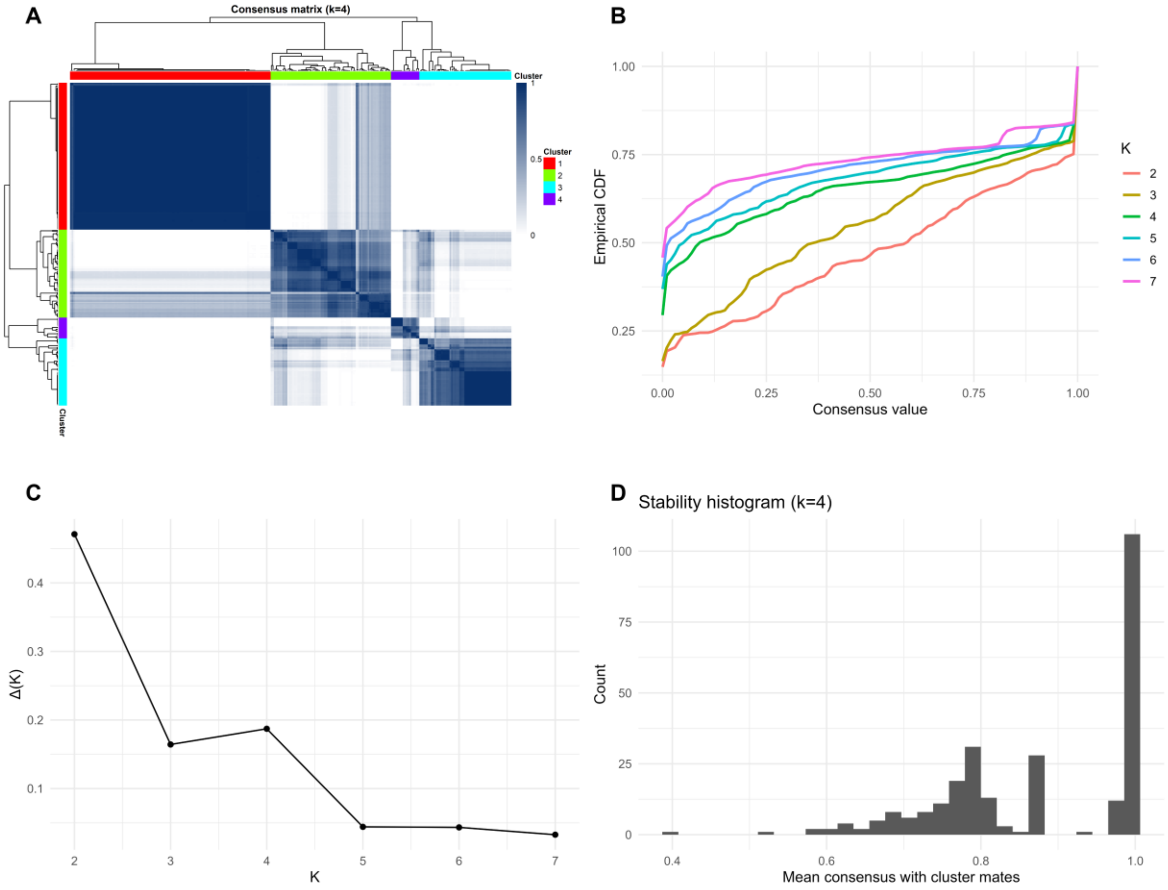

Figure S10. Consensus clustering diagnostic plots for neighboring second positions and four-fold synonymous sites in *gtsD* (n = 264). Results are shown for a random 80% subsample of the total number of four-fold synonymous sites and second positions within the gene; subsampling of each site type was performed independently. Plots are displayed for K = 4 as this cluster number is supported by consensus clustering (Table S8). (A) Consensus matrix heatmap. (B) CDF curve. (C) ΔK plot. (D) Site-level stability distribution.

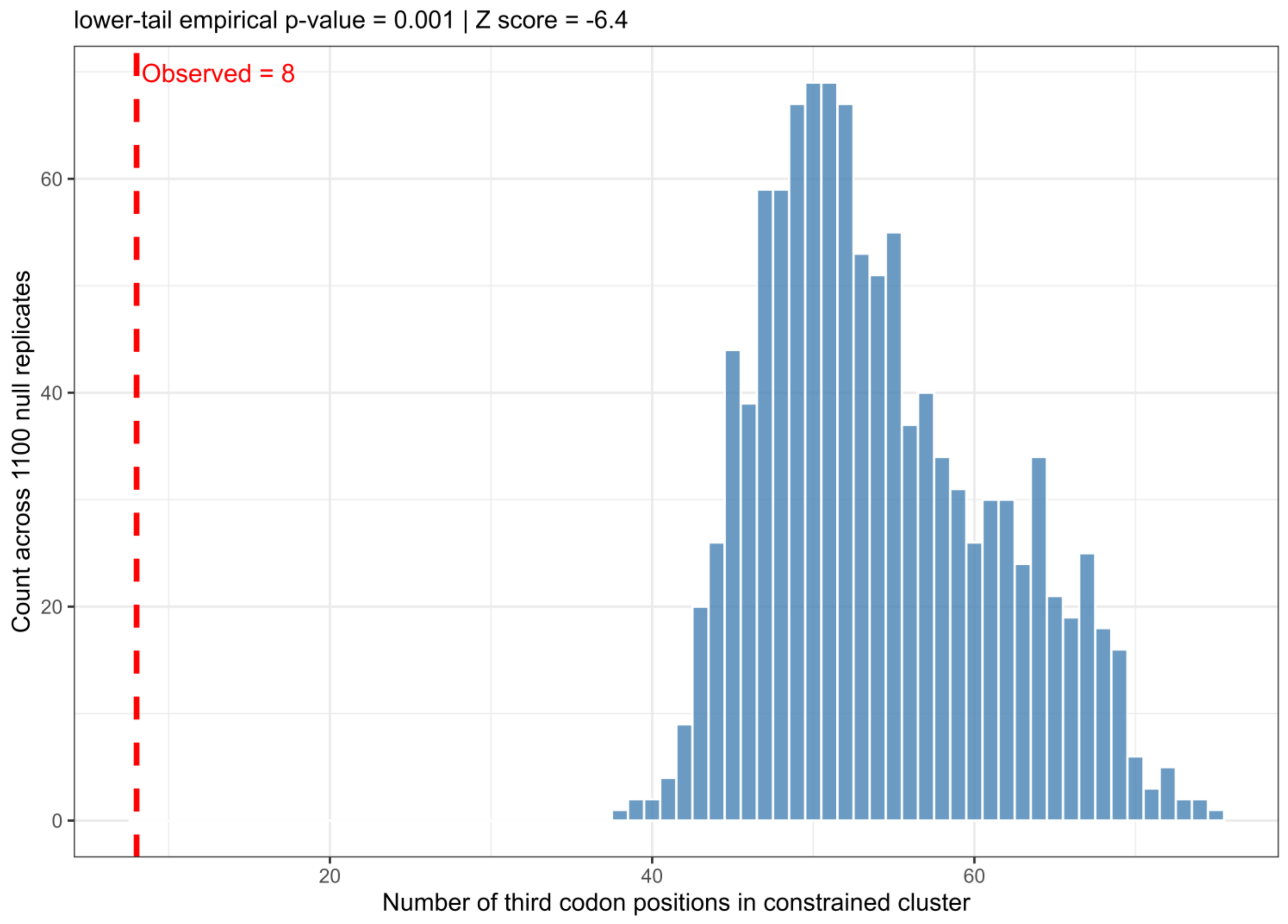

Figure S11. Empirical null distribution of third codon four-fold synonymous sites for *gtsB*. Histograms show the number of third codon four-fold positions assigned to the constrained cluster across 1100 permuted posterior predictive mappings. Red dashed vertical lines indicate the observed number of constrained synonymous sites in the real data (n = 8). The observed counts fall below the minimum value across all permuted mappings (lower-tail empirical p-value < 0.001).

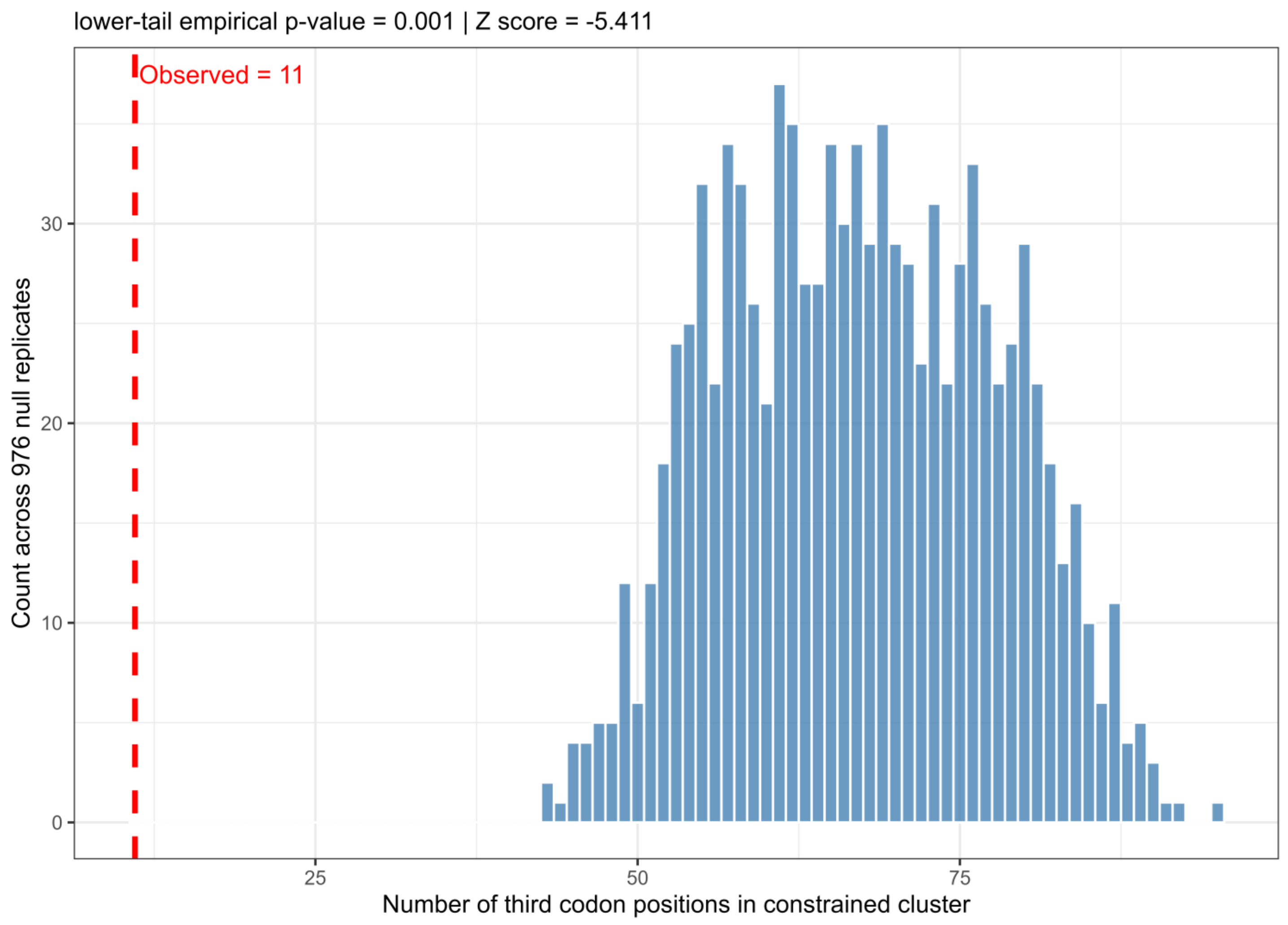

Figure S12. Empirical null distribution of third codon four-fold synonymous sites for *gtsD*. Histograms show the number of third four-fold codon positions assigned to the constrained cluster across 976 permuted posterior predictive mappings. Red dashed vertical lines indicate the observed number of constrained synonymous sites in the real data (n = 11 before consensus clustering assessment). The observed counts fall below the minimum value across all permuted mappings (lower-tail empirical p-value < 0.001).

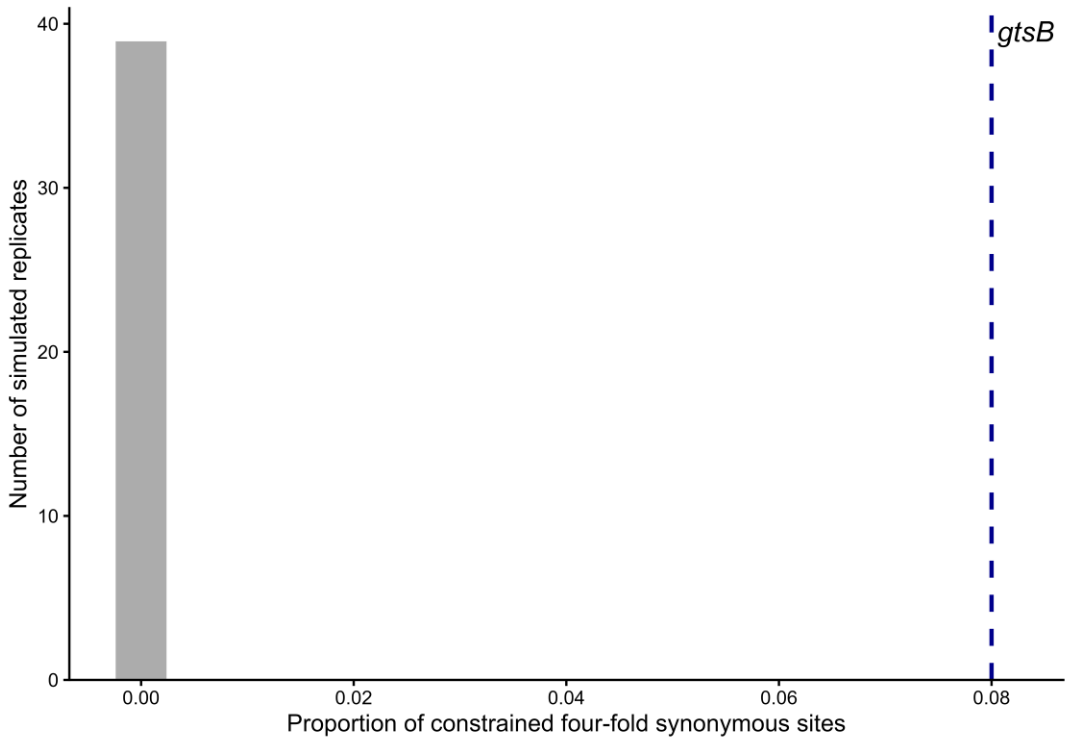

Figure S13. Proportion of 4-fold synonymous sites clustered with neighboring nonsynonymous sites across null replicates, compared to the observed proportion in *gtsB*. Total number of replicates = 39. The total number of synonymous sites among replicates ranged from 63 to 106. Under the null, the first two PCs, on average, collectively explained 63.26% of the variance (min = 60.86%, max = 66.63%, Table S15). Cluster and site stability were assessed using consensus clustering across three subsampling fractions (0.7, 0.8, and 0.9) and K = 2 to K = 7.

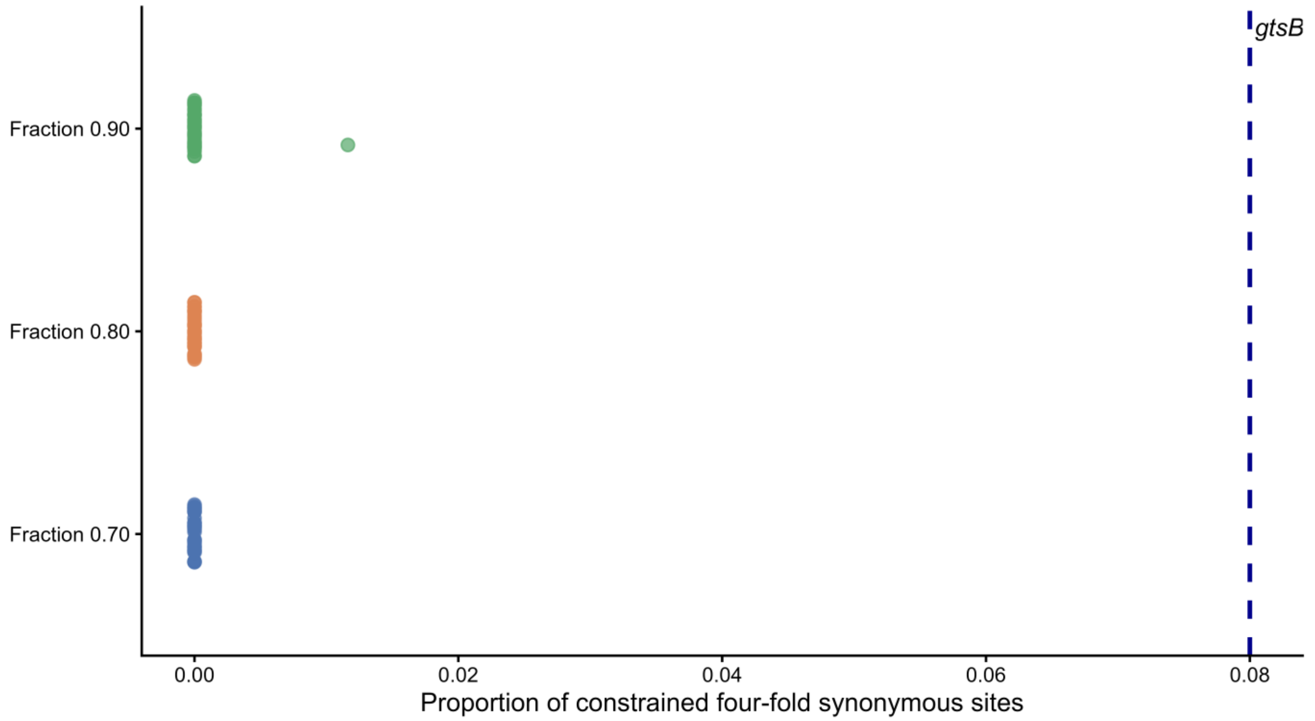

Figure S14. Proportion of 4-fold synonymous sites clustered with neighboring nonsynonymous sites across null replicates at three subsampling fractions, compared to the observed proportion in *gtsB*. Total number of replicates = 39. Dots correspond to the proportion of four-fold synonymous sites that were consistently assigned to the constrained cluster from K = 2 to K = 7 in each replicate and fraction.

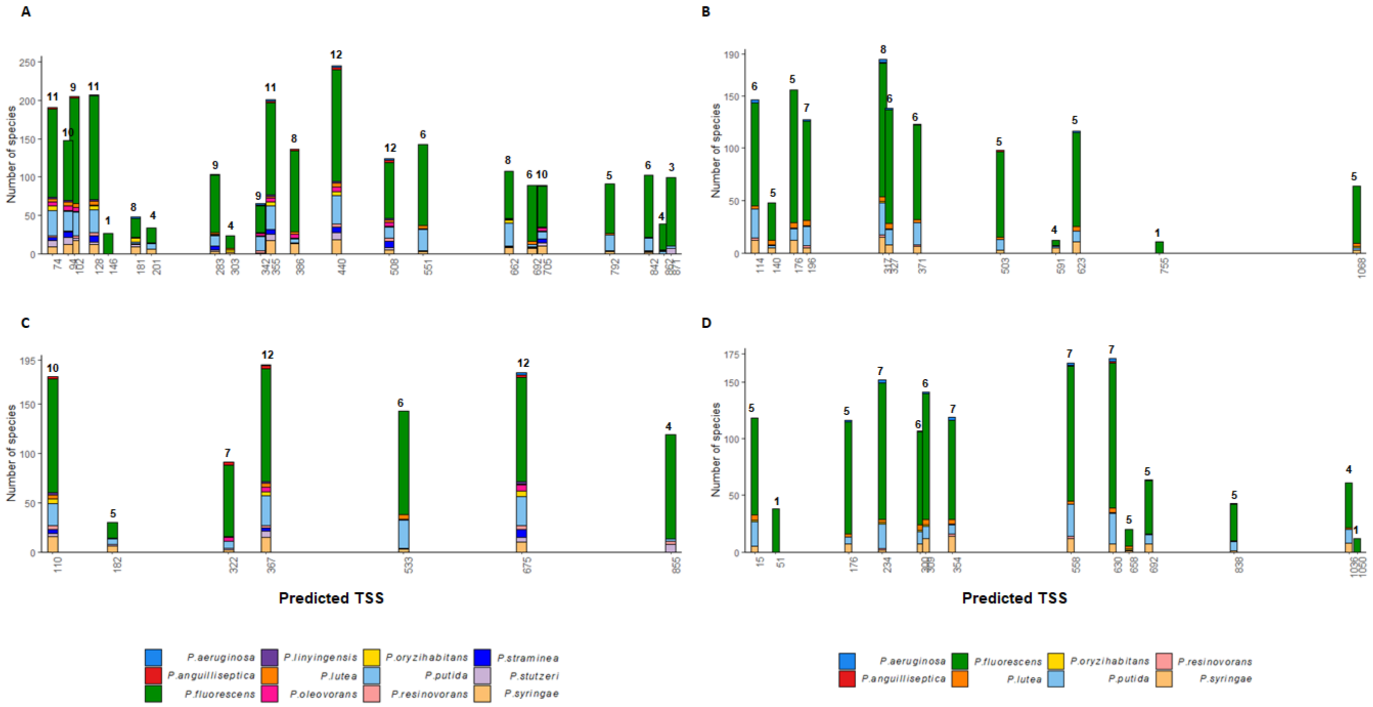

Figure S15. Repeatability of Transcription Start Sites (TSS) predicted in the *gtsB* and *gtsD* genes in *Pseudomonas fluorescens* SBW25 across a Pseudomonas phylogeny. (A) 22 TSS predicted on *gtsB* ORF. (B) 12 TSS predicted on *gtsD* ORF. (C) 7 TSS predicted in the reverse complement of *gtsB* ORF. (D) 14 TSS predicted on the reverse complement of *gtsD* ORF. Each color in the stack bars corresponds to the species within one of the 12 groups of Pseudomonas (N = 251 species in *gtsB* and N = 193 species *gtsD*). Numbers displayed at the top of each bar represent the number of Pseudomonas groups (colors). A TSS prediction was deemed to be present if it corresponded to the TSS prediction for P. fluorescens SBW25, or if it was predicted one position either preceding or following the SBW25-predicted site.

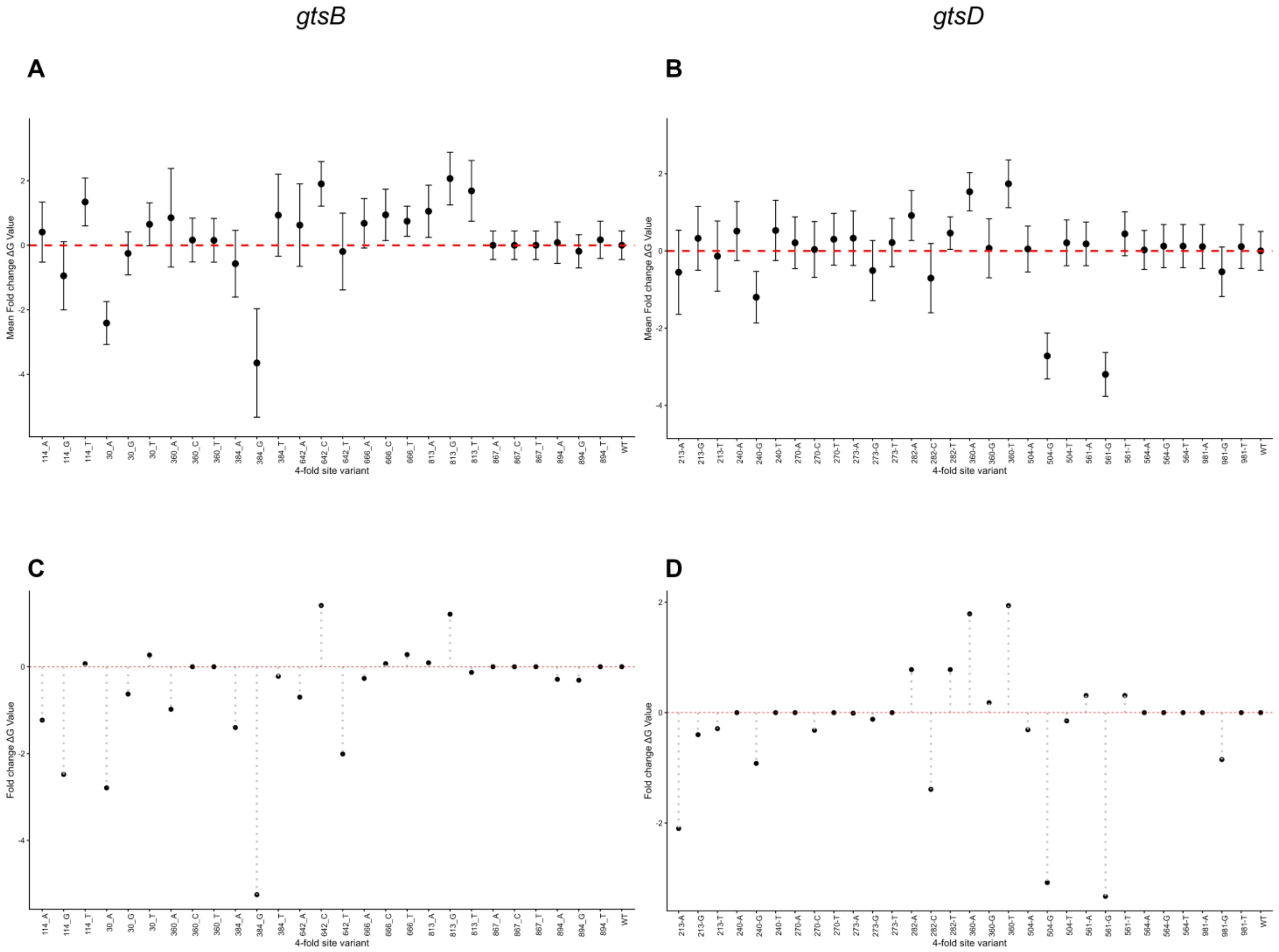

Figure S16. The predicted effect of four-fold alleles at identified constrained sites on mRNA folding stability, measured as free energy (kcal/mol), in the *gtsB* and *gtsD* sequences of *P. fluorescens* SBW25. A. Change in the first ten predicted structures in *gtsB* (n = 8) and B. Change in the first ten predicted structures in *gtsD* (n = 10). Error bars represent the standard error of the Mean. C-D. Change in the free energy on the first predicted structure of *gtsB* and *gtsD*, respectively.

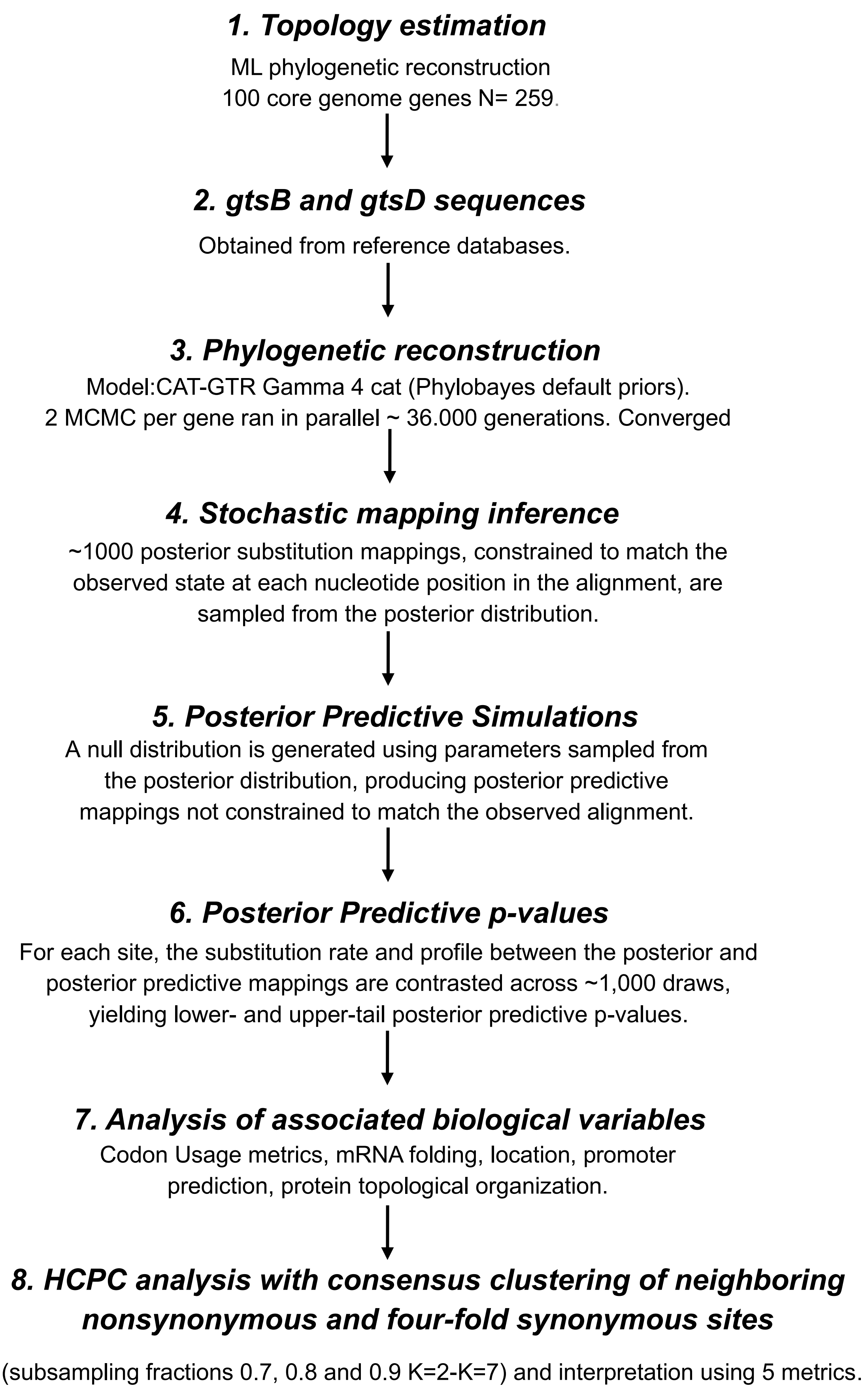

Figure S17. Summary of the methodological workflow followed in this study.
